# Defective Trophoblast Differentiation, Endothelial Dysfunction, and Immune Dysregulation in Preeclampsia Coalesce on a Placental VGLL3-Centered Gene Network

**DOI:** 10.1161/CIRCULATIONAHA.125.076218

**Published:** 2026-04-09

**Authors:** Olesya Plazyo, Laura B. Chopp, Rishyanth Peela, Kelly Young, Haihan Zhang, Rachael Bogle, Ashley Hesson, Elizabeth S. Langen, Ingrid L. Bergin, Li-Jyun Syu, Jake Erba, Joseph Kirma, Poulami Dey, Lin Zhang, Mrinal K. Sarkar, William R. Swindell, Katherine A. Gallagher, Nicole L. Ward, Kanakadurga Singer, J. Michelle Kahlenberg, Allison C. Billi, Andrzej A. Dlugosz, Santhi K. Ganesh, Lam C. Tsoi, Johann E. Gudjonsson

**Affiliations:** 1 Departments of Dermatology (O.P., L.B.C., R.P., K.Y., H.Z., R.B., L.-J.S., J.E., J.K., P.D., L.Z., M.K.S., J.M.K., A.C.B., A.A.D., L.C.T., J.E.G.), University of Michigan, Ann Arbor, MI.; 2 Obstetrics(and)Gynecology (A.H., E.S.L.), University of Michigan, Ann Arbor, MI.; 3 Cell(and)Developmental Biology (L.-J.S., A.A.D.), University of Michigan, Ann Arbor, MI.; 4 Surgery (K.A.G.), University of Michigan, Ann Arbor, MI.; 5 Pediatrics (K.S.), University of Michigan, Ann Arbor, MI.; 6 Internal Medicine (J.M.K., S.K.G., J.E.G.), University of Michigan, Ann Arbor, MI.; 7 Human Genetics (S.K.G.), University of Michigan, Ann Arbor, MI.; 8 Biostatistics (L.C.T.), University of Michigan, Ann Arbor, MI.; 9 Computational Medicine(and)Bioinformatics (L.C.T.), University of Michigan, Ann Arbor, MI.; 10 Unit for Laboratory Animal Medicine (I.L.B.), University of Michigan, Ann Arbor, MI.; 11 Department of Internal Medicine, UT Southwestern Medical Center, Dallas, TX (W.R.S.).; 12 Department of Dermatology, Vanderbilt University Medical Center, Nashville, TN (N.L.W.).

**Keywords:** endothelium, vascular, genes, regulator, Hippo signaling pathway, hypertension, pregnancy induced, immunity, active, pre-eclampsia, trophoblasts

## Abstract

**BACKGROUND:**

Preeclampsia affects approximately 1 in 10 pregnancies, leading to severe complications and long-term health risks for both mother and offspring. While the etiology remains unclear, preeclampsia has been linked to both autoimmunity and the timing of menarche.

**METHODS:**

Through human single-cell and spatial analyses, coupled with in vitro, in vivo, and ex vivo models, we demonstrate that VGLL3 (Vestigial-like family member 3), a transcription coregulator in the Hippo pathway, is upregulated in preeclamptic placentas.

**RESULTS:**

VGLL3 promotes immune activation, impairs trophoblast differentiation, and induces endothelial dysfunction, all of which contribute to pregnancy-related hypertension, fetal growth restriction, and offspring mortality. Our data reveal that VGLL3 acts upstream of preeclampsia-associated processes, including the production of sFLT1 (soluble fms-like tyrosine kinase 1), a key biomarker of the disease. Notably, targeting VGLL3, either by genetic deletion in mouse placentas or through therapeutic inhibition in human placentas, protects against preeclampsia and alleviates disease pathology.

**CONCLUSIONS:**

These findings position VGLL3 as a promising novel therapeutic target for preeclampsia.

**Clinical Perspective**

**What Is New?**

- The Hippo pathway is strikingly dysregulated in preeclampsia (PreE), with increased expression and enhanced colocalization of TEAD transcription factors and VGLL3 in placental trophoblasts.
- VGLL3 drives a pathogenic transcriptional program in PreE, altering trophoblast differentiation, promoting a proinflammatory microenvironment, and contributing to endothelial dysfunction.
- Placenta-specific overexpression of *Vgll3* in mice reproduces core features of PreE, including maternal hypertension, and increases neonatal mortality, establishing VGLL3 as a causal driver of disease.

**What Are the Clinical Implications?**

- Pharmacologic inhibition of the Hippo pathway in human placental explants reverses PreE-associated gene expression, while mice lacking *Vgll3* experience normal pregnancies, supporting VGLL3 as a tractable therapeutic target.
- Ongoing efforts aim to develop strategies for selectively targeting placental VGLL3 as a novel treatment approach for PreE.
- VGLL3 mechanistically links PreE with autoimmune disease; thus, patients with autoimmune disease may benefit from enhanced surveillance for the development of PreE.

Preeclampsia (PreE) is a vascular endothelial disorder of pregnancy accompanied by hypertension and associated with severe perinatal morbidity for mother and child, including death and lifelong complications.^1^ Due to the limited understanding of PreE pathogenesis, US Food and Drug Administration–approved treatment options remain scarce, with delivery of the fetus and placenta serving as the only definitive intervention.

Previous studies have broadly categorized the major pathological mechanisms underlying PreE into 4 key areas: placental dysfunction (including inadequate spiral artery remodeling and a shift toward antiangiogenic signaling), metabolic disturbances, maternal antifetal immune rejection, and dysregulation of extracellular matrix proteins.^2^ An overarching mechanism involves proinflammatory cytokines released from aberrantly activated innate and adaptive immune cells, including macrophages and T cells, along with antiangiogenic factors released from trophoblasts.^3^

A hallmark of PreE is increased placental secretion of soluble fms-like tyrosine kinase 1 (sFLT1)/VEGFR1 (vascular endothelial growth factor receptor 1)^4^ and reduced production of PlGF (placental growth factor).^5^

The ratio of these 2 markers currently forms the basis of the only Food and Drug Administration–approved test for assessing PreE risk.^5^ Although therapeutic strategies targeting sFLT1 have shown promise in preclinical animal models,^6^ their effectiveness in human patients remains under investigation. Deciphering the complex etiology of PreE is essential for identifying additional therapeutic targets.

Using single-cell and spatial transcriptomics alongside human placental explants, mouse models, and in vitro systems, we show that PreE molecular features converge on a VGLL3-centered network connecting its core pathological mechanisms with its association with autoimmunity^7,8^ and early menarche.^9,10^

## METHODS

The data, methods used in the analysis, and materials used to conduct the research are available to any researcher for purposes of reproducing the results or replicating the procedure.

### Human Placentas

The University of Michigan institutional review board has approved collection of human placentas for this study (HUM00184982). All patients gave written consent. Patient demographics data are listed in Table 1. Severe PreE features were defined according the American College of Obstetricians and Gynecologists Task Force report on hypertension in pregnancy.^11^ Cases with choroamnionitis, infections, chronic hypertension, and nonsingleton pregnancies were excluded. Immediately following delivery, whole-thickness placental samples and chorioamniotic membranes were digested using the Miltenyi Biotec Umbilical Cord Dissociation Kit to obtain a single-cell suspension according to the manufacturer’s protocol. Briefly, tissues were washed, cut into small (2–3 mm) pieces with sterile scissors or a blade, incubated with a digestive enzyme cocktail for 2 hours at 37 °C, and dissociated using a gentleMACS dissociator running the spleen_01 program. Samples were then run through 100-µm strainers, incubated with red blood cell lysis buffer (Biolegend), washed, and resuspended in Iscove’s Modified Dulbecco’s Medium supplemented with 10% fetal bovine serum for delivery to the University of Michigan (UM) Advanced Genomics Core for single-cell RNA sequencing (scRNA-seq). Cell viability was confirmed to be above 80% for all samples. Libraries were constructed on the 10× Chromium system with chemistry v3 and sequenced on the Illumina NovaSeq 6000 sequencer to generate 150-bp paired-end reads.

**Table 1.**
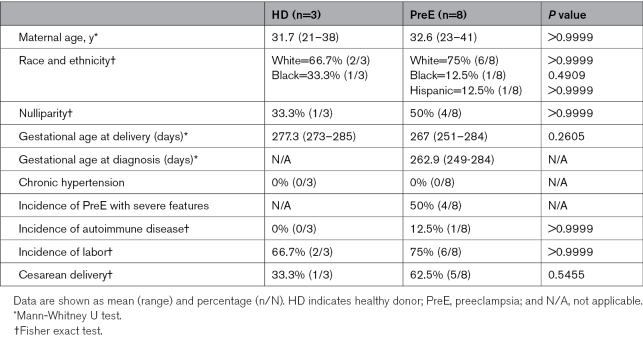
Demographics of Donors Whose Placentas Were Used for scRNA-seq Analyses

### scRNA-seq Analyses

Data were preprocessed in Seurat^12^ by removing genes expressed in fewer than 2 cells and excluding cells that were outliers for number of RNA molecules or more than 10% mitochondrial genes. DoubletFinder^13^ was used to identify and remove probable doublet cells (principal component 1:15, proportion of artificial doublets default 0.25, number of expected doublets was determined with estimated doublet on the basis of loading density for the 10× platform), and DecontX^14^ was used to remove probable RNA contamination from individual cells. The individual samples were merged into 1 object and preprocessed with the standard Seurat analysis, including normalization, variable feature identification, data scaling, and principal component analysis (PCA). The samples were integrated using the RunHarmony^15^ command in Seurat to mitigate batch effects. Uniform manifold approximation and projection (UMAP) dimensional reduction was done using the RunUMAP function in Seurat. Unsupervised clustering was performed by identifying the nearest neighbors using the first 20 dimensions and identifying clusters (resolutions used: human untreated, 0.1; human inhibitor treated, 0.5; mouse Vgll3 overexpressing [OE], 0.3; mouse lipopolysaccharide [LPS] treated, 0.3). The top cluster-defining genes were found using the FindAllMarkers function (absolute log2 fold change threshold of 0.5), and these cluster-defining genes were used to annotate the cell types on the basis of canonical gene signatures and previously published data.^16^ PreE-specific genes were identified by using FindMarkers to identify differentially expressed genes between healthy donors (HDs) and PreE on total placentas with a minimum log2 fold change threshold of +0.1. Putative upstream regulators of this gene signature were identified using Ingenuity Pathway Analysis and plotted on a volcano plot showing the activation *Z* score and false discovery rate. The proportion of *VGLL3*-expressing cells was calculated by taking the number of *VGLL3*+ cells in each individual cluster divided by the total number of cells within the dataset. To perform higher-resolution analysis of endothelial and trophoblast clusters, extravillous trophoblasts (EVTs), endovascular EVTs, vascular smooth muscle (VSM) cells, immunomodulatory endothelial cells, lymphatic endothelial cells, and capillary endothelial cells were subsetted into a Seurat object and reprocessed, including normalization, variable feature identification, data scaling, and principal component analysis identification. Clustering was performed with a resolution of 0.2.

### Gene Signature Definition and Scoring

The composition of the Hippo pathway–related genes and fibrosis signature are shown in Table S4. PreE-specific gene signatures were identified with the FindMarker command on individual clusters with a minimum log2 fold change threshold of +0.1 and *P*<0.05. The AddModuleScore function was used to score individual cells for these signatures (control = 10). To score PreE gene signatures on mouse data, mouse orthologs of signatures were identified and scored. *Vgll3*-OE–specific genes were identified with the FindMarker command on individual clusters with a minimum log2 fold change threshold of +0.1, minimum percentage expressed of 10%, and *P*<0.05. Human orthologs of these signatures were scored on the human dataset with the AddModuleScore function.

### Pseudotime Trajectory Construction

Trophoblast clusters (cytotrophoblasts [CTBs], transitional trophoblasts, syncytiotrophoblasts [STBs], and EVTs) were subsetted into a Seurat object and separated by disease state. These objects were imported into Monocle3 by generating a cell dataset from the decontaminated counts slot. Normalization and principal component analysis were done with the preprocess_cds command from Monocle3, with the first 100 dimensions using log normalization, and batch correction was performed using the align_cds command with continuous effects, which uses the Batchelor tool.^17^ Uniform manifold approximation and projection dimensional reduction was performed using the reduce_dimension command. Cells were clustered with the cluster_cells command with default parameters. The trajectory graph was learned on the Monocle-derived clusters with learn_graph. Cells on the uniform manifold approximation and projection plot are colored by Seurat-derived clusters.

Pseudotime was determined using CTBs as the starting point. Genes that increase with pseudotime in either the STB or EVT lineage were identified using the find_gene_modules function and selecting the modules that increased expression in either lineage. These gene lists were then filtered for genes with higher expression in PreE than HD cells. Ingenuity Pathway Analysis was then used to identify putative upstream regulators of these signatures.

### Ligand-Receptor Interaction Analyses

CellphoneDB (v4.1.0) was used to identify putative ligand-receptor interactions from scRNA-seq datasets.^18^ To identify interactions between *VGLL3*+ EVT cells and other cell types in PreE placentas, a new cell identity was created to denote cells that were positive or negative for *VGLL3* expression. Interactions with *P*<0.05 were retained, and the number of interactions was plotted with ktplots on a heatmap and on a bar chart. Select interactions were plotted on dot plots showing the strength of the interaction. Mouse *Vgll3*-OE and LPS-treated control versus *Vgll3* knockout (KO) analyses were performed on each genotype separately, and then select interactions were shown on dot plots. Inhibitor-treated human samples were run separately and significant interactions retained. A summation of all interactions was generated and plotted as a heatmap for each condition. Ligand–receptor interactions that were mutually expressed within the same cell types between the conditions were removed, and the resulting nonoverlapping interactions were also plotted as a heatmap. Ligands and receptors that were associated with a cytokine, complement, or Hippo pathway were plotted as Circos plots, also removing mutual interactions between conditions.

### Spatial Sequencing

Formalin-fixed, paraffin-embedded (FFPE) placentas from a PreE patient and HD were re-embedded into a single block, sectioned onto a slide, and subjected to Xenium in situ transcriptomics preparation and sequencing at the UM Advanced Genomics Core. For data analyses, we initially employed Symphony^19^ to annotate our spatial Xenium dataset by mapping it onto a well-curated, annotated scRNA-seq reference obtained from the current work. This approach enabled robust and accurate cell type identification. Subsequently, we utilized the R package Giotto^20^ to generate in situ plots that visually represent both the cell type annotations and the spatial expression patterns of targeted genes. Finally, differential gene expression analysis was performed using scran^21^ as implemented within the Giotto framework, facilitating the robust identification of gene expression differences across distinct cellular populations.

### Double-Axis Scatterplots

Differentially expressed genes (DEGs) significantly changed (*P*<0.05) between treatment and control groups for each cell type of interest were entered into Enrichr to assess pathway enrichment. The compiled pathway data facilitated identification of the pathways shared among all cell types, with at least the top 3 pathways for each cell type chosen for representation. The double-axis scatterplots were created in Tableau with “Cell Types” as columns, “Pathways” as rows, and the intersection of these 2 variables as the negative log *P* values, with larger (more significant) values corresponding to heavier dots in the scatterplot.

### Bulk RNA-seq

Five 20-µm sections per sample were obtained from FFPE biopsies. RNA isolation was performed using the QIAGEN RNeasy FFPE Kit (73504). In brief, sections were transferred to a microcentrifuge tube treated with QIAGEN deparaffinization solution and briefly incubated at 56 °C. Following a subsequent incubation in a proteinase K lysis buffer at 56 °C, tissue sections were shifted to a higher temperature (80 °C) to partially reverse formalin crosslinking of the released nucleic acids. This was followed by DNase treatment optimized to eliminate all genomic DNA. The obtained lysates were mixed with buffer RBC and ethanol and subjected to an RNeasy MinElute spin column. The obtained RNA was eluted with 30 µL of RNase-free water. Libraries were prepared using the QuantSeq 3′ mRNA-Seq Library Prep Kit and sequenced on Illumina NovaSeq 6000 SP Flow Cells. For RNA-Seq analyses, adapter trimming and quality control were conducted on the raw sequence reads. The paired-end reads were mapped using Spliced Transcripts Alignment to a Reference^22^ to Genome Reference Consortium Human Build 37. Only uniquely mapped reads were used for subsequent analysis. Gene expression levels were quantified with GENCODE v24 used as a reference and normalized by HTSeq^23^ and DESeq2.^24^

### Immunohistochemistry/Proximity Ligation Assay

FFPE human and mouse tissue sections were heated at 60 °C for 30 minutes, deparaffinized, and rehydrated. Slides were placed in either pH 9 or pH 6 antigen retrieval buffer, depending on the antibody specification. Slides were cooled in ice, treated with 3% H_2_O_2_ for 5 minutes, and blocked with 10% serum for 30 minutes. Primary antibodies included the following: Neutrophil elastase (Abcam, ab310335), CD3 (Abcam, ab215212), CD19 (Thermo Fisher Scientific, pa5-27442), VGLL3 (Sigma, HPA054983), FLT1 (Abcam, ab2350), TEAD1 (Santa Cruz Biotechnology, sc-393976), TEAD2 (LS Bio, LS-C342577), TEAD3 (Creative Biolabs, CBMAB-0234-LY), and TEAD4 (Santa Cruz Biotechnology, sc-390578). After overnight incubation at 4 °C, sections were washed and incubated with a secondary antibody for 1 hour at room temperature. Following counterstaining with hematoxylin and bluing reagent, slides were imaged under a Carl Zeiss Axioskop 2 microscope. For the proximity ligation assay, 5-µm sections were prepared from FFPE human placenta samples and stained using the Duolink Proximity Ligation Assay Kit according to the manufacturer’s instructions (Sigma-Aldrich).

### Cell Culture, Transfection, Immunoprecipitation, and Protein Analyses

HTR8 cells were cultured in Gibco RPMI 1640 medium (Invitrogen) with 10% fetal bovine serum. HEK293 cells were grown in Gibco high-glucose DMEM (Invitrogen) with 10% fetal bovine serum. Cells were grown to over 70% confluence and then transfected using Lipofectamine 3000 (Thermo Fisher Scientific) according to the manufacturer’s protocol to express Myc-DDK-tagged VGLL3 in its native form (OriGene, RC218896) or mutagenized to preclude VGLL3-TEAD binding. For immunoprecipitation, 500 µg to 1 mg of total protein was incubated overnight with an anti-FLAG antibody (OriGene, TA50011) on a shaker at 4 °C. Protein A–agarose was used to pull down immune complexes, which were then analyzed by immunoblotting and mass spectrometry. For immunoblotting, proteins were denatured in sample buffer containing β-mercaptoethanol and boiled at 100 °C for 3 min. Samples were separated on gradient 4% to 20% SDS-PAGE gels (Thermo Fisher Scientific) and electroblotted to nitrocellulose. Primary antibodies included the following: TEAD1 (OriGene, RG215492), GFP (OriGene, TA150041), FLAG (Origene, TA50011), and VGLL3 (Sigma, HPA054983). Bands were detected on the iBright system using enhanced chemiluminescence substrate. Liquid chromatography-tandem mass spectrometry was performed at the UM Proteomics Resource Facility. For RNA interference, small interfering RNA (siRNA) was introduced either by electroporation using Lonza 4D-nucleofector following the manufacturer’s instructions as described previously^25^ or using self-delivering Dharmacon Accell siRNA (Horizon Discovery). Nontargeting siRNA was use as a negative control. RNA was harvested with the QIAGEN RNeasy Plus Kit, and RNA-seq libraries were prepared using Illumina Truseq RNA Library Prep Kits.

### Design and Validation of Cre-Inducible *Vgll3* Mice

All mouse procedures were performed in accordance with institutional guidelines. The *Rosa26* locus on chromosome 6, a “safe harbor” genomic site, was used for the integration of mouse *Vgll3* downstream of a floxed EGFP with a STOP sequence to achieve conditional gene expression in mice. Mouse *Vgll3* with *IRES* DNA (1560 bp) was polymerase chain reaction (PCR) amplified from *K5-Vgll3-IRES-mCherry*, the construct used for generating our previous K5 promoter-driven *Vgll3* transgenic mouse model.^26^ The PCR fragment was then cloned into a previously generated *CAG-loxP-EGFP-STOP-loxP-rtTA3-P2A-mCherry-SV40-polyA* cassette, where it replaced *rtTA3-P2A*. This cassette was built by replacing the sequence from CAG up to the *Rosa26* right arm in the backbone of the pR26 CAG AsiSI/MluI plasmid^27^ (Addgene, 74286). We designed cloning protocols that were carried out by GenScript. The 15.5-kb plasmid was knocked into the mouse *Rosa26* locus of fertilized oocytes using the CRISPR-Cas9 system by the UM Transgenic Animal Model Core. Three transgenic mouse lines were validated by analyzing high-molecular-weight genomic DNAs extracted from F1 pups with long-range PCR (New England Biolabs, M0323S LongAmp *Tag* DNA polymerase), to confirm proper 5′ and 3′ integration into the *Rosa26* locus. Two thousand fifty-eight base pairs of 5′ flanking sequences were PCR amplified using a primer pair of 5′-CTAGGTAGGGGATCGGGACTCTG and 5′-AGTAGGAAAGTCCCATAAGGTC (targeting the cytomegalovirus promoter). Five thousand ninety-three base pairs of 3′ flanking sequences were PCR amplified using a primer pair of 5′-CACCATCGTGGAACAGTACGAAC (targeting mCherry) and 5′-CAGTGGCTCAACAACACTTGGTC.

### Mouse Breeding and Phenotyping

*Cyp19-Cre* males were time mated with *R26-LSL-Vgll3* females to induce *Vgll3* overexpression specifically in the placenta. Detection of a vaginal plug in the morning following coitus was designated as embryonic day (E) 0.5. Blood pressure monitoring was performed at E16.5 using a tail-cuff method on CODA Noninvasive Blood Pressure Monitoring System (Kent Scientific) at the UM Physiology Phenotyping Core. Genotyping was carried out by the Transnetyx automated service using custom-designed probes. Placental histological examinations were conducted by a pathologist on 10 hematoxylin–eosin–stained sections of murine placentas at the Unit for Laboratory Animal Medicine Pathology Core, where complete blood count and serum chemistries were also performed. PlGF in maternal circulation was measured by ELISA according to the manufacturer’s protocol (LS Bio, LS-F5507-1). Ejection fraction was measured at E15.5 during ultrasound scanning with Echo at the UM Physiology Phenotypic Core. For LPS administration (Sigma-Aldrich, L2630), intraperitoneal injections were performed daily for 10 days starting at E7.5, as described previously,^28^ and placentas were harvested at E18.5.

*Vgll3* KO mice (strain ID EM:11403, C57BL/6NTac-Vgll3/H) were obtained from the Medical Research Council Harwell Institute. They were generated by CRISPR-induced deletion of 890 nt from the *Vgll3* gene, including a critical exon 2 ENSMUSE00000876808, to induce a premature stop codon and a null allele.

Blinding and randomization were performed in all mouse experiments when feasible.

### Animal Body Composition Analysis

Body fat, lean mass, free water, and total water were quantified using an NMR-based EchoMRI 4in1-500 analyzer. Conscious mice were placed individually in a clear plastic holder and inserted into the analyzer without anesthesia or sedation, allowing for noninvasive, rapid measurements within 2 minutes. The EchoMRI system differentiates tissue composition by exploiting differences in relaxation times of hydrogen proton spins, generating high-contrast signals through specialized radio pulse sequences. Calibration was performed daily using a canola oil reference sample according to the manufacturer’s recommendations.

### Verteporfin Treatment

Fresh, whole-thickness placentas and chorioamniotic membranes obtained from patients with PreE were minced with a sterile blade, washed in PBS, and incubated with 10 µM verteporfin (Cayman Chemical, 17334) or DMSO as a vehicle control at 4 °C overnight. After centrifugation, single-cell suspensions were obtained by enzymatic digestion for scRNA-seq as described for the untreated placentas.

### Statistical Analysis

All RNA-seq analyses were performed using statistical frameworks that incorporate false discovery rate correction, which substantially mitigates false positives in studies with modest sample sizes. In addition, our differential expression analyses used established tools (DESeq2/edgeR/Seurat; see details under Bulk RNA-seq) that explicitly model variance across genes to control for type I error. For bulk RNA-seq, our sample size aligns with prior studies in the field, and power calculations based on observed effect sizes indicate adequate power to detect biologically meaningful differences. For scRNA-seq, the large number of cells provides substantial statistical power at the cellular level, even when the number of donors is limited, and we relied on pseudobulk and mixed-model approaches where appropriate to ensure robust inference. No repeat samples were taken from the same animal; each data point represents a biologically independent sample derived from a distinct animal. To detect subtle changes in gene expression, raw unadjusted *P* values were used in some analyses. For datasets with sufficient sample size, normality was evaluated using the Shapiro–Wilk test. For mouse studies and other experiments with small sample sizes (n<10), where normality cannot be reliably assessed, we used the nonparametric Mann–Whitney U test. Specific tests can be found in the figure legends. Multiple testing corrections were applied using an adjustment method indicated in each figure (e.g., false discovery rate/Benjamini-Hochberg). Pathway analyses were not subjected to multiple testing corrections, which is a limitation of the study.

### Resource Availability

Requests for further information and resources should be directed to and will be fulfilled by the corresponding author.

Mouse lines and any other unique/stable reagents generated in this study will be made available upon request.

### Data and Code Availability

Sequencing data have been deposited at the NCBI Gene Expression Omnibus as GSE298602 and are publicly available as of the date of publication.

## RESULTS

### scRNA-seq and Spatial RNA-seq Uncover Coordinated Immune Activation, Altered Stromal and Trophoblast Signaling, and Hippo Pathway Dysregulation in PreE

We performed scRNA-seq on HDs (n=3) and individuals with PreE (n=8) (Table 1), generating a dataset of 45 813 high-quality cells.

**Figure 1.**
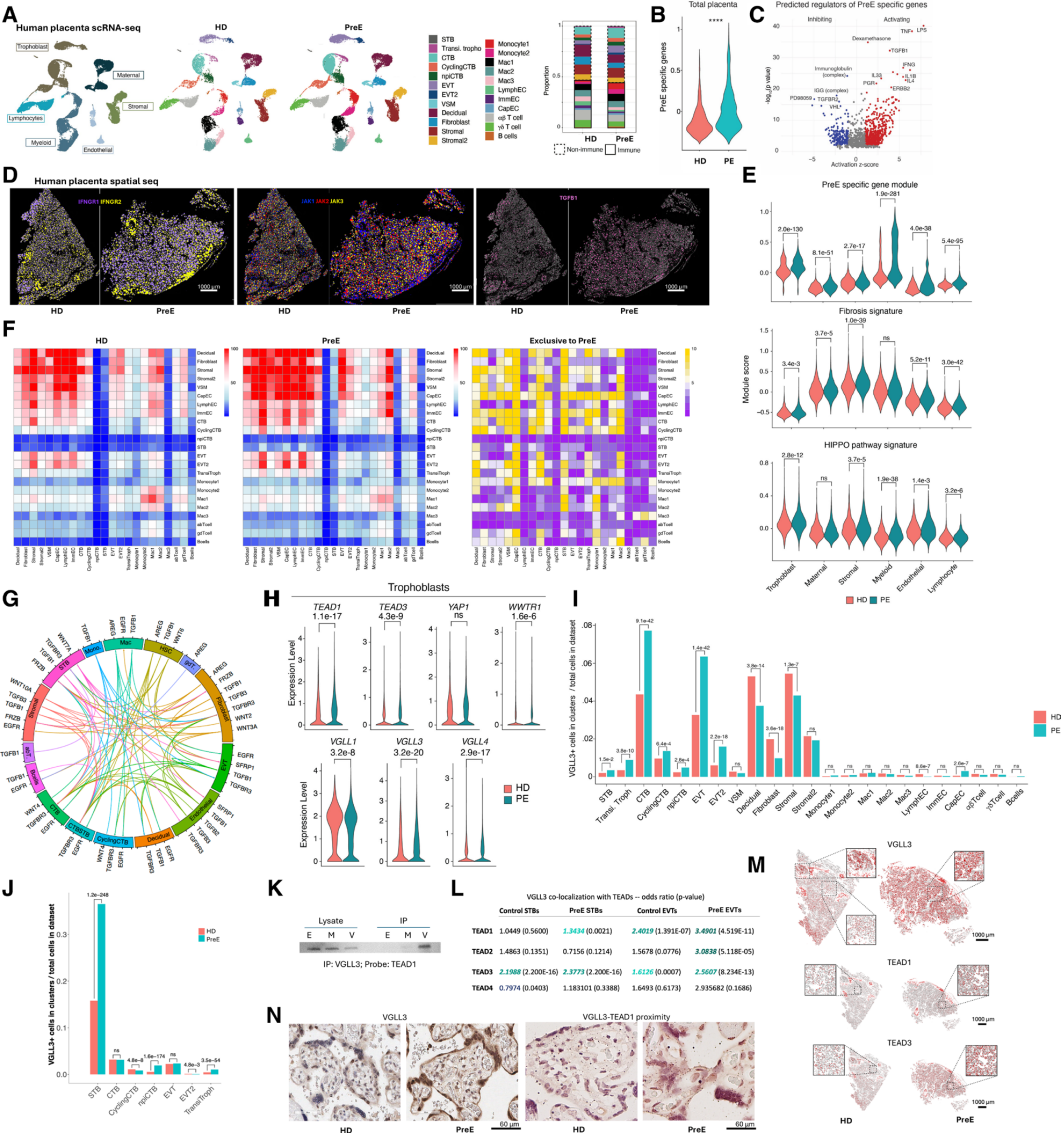
**Single-cell RNA sequencing (scRNA-seq) and spatial RNA-seq implicate the Hippo pathway in preeclampsia (PreE). A**, Uniform manifold approximation and projection plot showing placental cells from healthy donors (HDs) or patients with PreE, colored by main cell type (**left**) and their subtypes (**middle**) and bar plot showing the proportion of clusters in each condition (**right**, same color coding). **B**, Violin plot showing the module score of PreE-specific genes calculated on total placenta between conditions. Significance was calculated with a Wilcoxon rank sum test. ****P<2.2×10^−16^ was at the limit of R’s floating-point precision (2.225074^−308^) (**left**). **C**, Volcano plot showing putative regulators of this gene signature predicted by Ingenuity Pathway Analysis (activation *Z* score [*x* axis] vs *P* value, −log10 scale [*y* axis]). Red dots indicate activating regulator, blue dots indicate repressive regulator. Volcano plots show differential gene expression (log2 fold change [*x* axis] vs *P* value, −log10 scale [*y* axis]) between HD and PreE in the indicated myeloid clusters. **D**, expression patterns of IFNG receptors (**left**), JAK kinases (**middle**), and TGFB1 (**right**), determined by spatial sequencing. **E**, Gene module scores were calculated to assess PreE-, fibrosis-, and Hippo pathway–specific gene expression across main cell populations. Significance was calculated with a Wilcoxon rank sum test with Benjamini-Hochberg testing for multiple comparisons. Adjusted *P* values (false discovery rate) are shown above each plot. **F**, Ligand-receptor interaction analyses in healthy and PreE placentas allowed identification of PreE-specific cell-cell interactions. **G**, Circos plot of PreE-specific Hippo pathway receptor–ligand interactions in major cell types. **H**, Violin plots showing expression of main regulators in the Hippo pathway in HD and PreE trophoblasts. Significance was calculated with Wilcoxon rank sum test with Bonferroni correction. False discovery rate values are shown above each plot. **I**, bar plot showing proportion of VGLL3^+^ cells in individual clusters over total cells in the sample based on scRNA-seq data. Significance was calculated with a 2-sample test for equality of proportions with continuity correction, followed by Benjamini-Hochberg test for multiple comparisons. Adjusted *P* values are shown above each plot. **J**, bar plot showing proportion of VGLL3^+^ cells in individual clusters over total cells in the sample based on spatial sequencing data. Significance was calculated with Fisher exact test. *P* values are shown above each plot. **K**, Co-immunoprecipitation confirmed VGLL3-TEAD1 binding. HTR8 cells were transfected either with empty vector (E), mutated VGLL3 (M), or full-length wild-type VGLL3 (V). **L**, Colocalization of VGLL3 with TEADs in syncytiotrophoblasts (STBs) and extravillous trophoblasts (EVTs), computed from Xenium spatial sequencing. Odds ratios and *P* values obtained using Fisher exact test are shown. **M**, Expression pattern of VGLL3, TEAD1, and TEAD3, determined by spatial sequencing. **N**, Immunohistochemistry (**left**) confirmed increased expression of VGLL3 at the protein level in PreE placentas. Increased VGLL3-TEAD1 proximity in PreE placentas was confirmed by proximity ligation assay (**right**). Representative images are based on at least 3 independent experiments. CapEC indicates capillary endothelial cell; CTB, cytotrophoblast; EVT2, endovascular extravillous trophoblast; ImmEC, immunomodulatory endothelial cell; IP, immunoprecipitation; LymphEC, lymphatic endothelial cell; Mac, macrophages; npiCTB, non-proliferative interstitial cytotrophoblasts; Transi Tropho, transitional trophoblasts; and VSM, vascular smooth muscle.

Unsupervised clustering identified 23 cell states spanning trophoblasts, endothelial, lymphoid, and myeloid linages (Figure 1A; Table S1; Figure S1). Although major cell type proportions were broadly preserved, PreE placentas showed increased monocytes, B cells, and EVTs and decreased αβ T cells and Mac3 macrophages (Figure 1A, right).

Pseudobulk analysis revealed upregulation of PreE-associated genes (Figure 1B, left), and

Ingenuity Pathway Analysis implicated TNF, TGF-β1, IFNγ, IL-1B, IL-4, and IL-33 (Figure 1B, right) as upstream regulators, consistent with robust immune dysregulation.

#### Immune Cell Activation in PreE

Monocytes and macrophages displayed elevated expression of proinflammatory genes (e.g., *CXCL2*, *IL1A*, *CCL3*, and *CCL20*; Figure S2A), consistent with prior observations.^29–32^ T-cell subsets showed heightened inflammatory signatures, including *IL32* and *GZMA* in γδ T cells and *IFNG*, *GZMH*, and *GZMA* in CD8 T cells (Figure S2B), and altered signaling molecule expression in CD4 T cells (e.g., *FYN*, *JUNB*, and *CD40LG*), consistent with impaired regulatory function, as described previously.^33^ Although few in number, B cells showed reduced expression of *SPIB*, a transcription factor that limits plasma cell differentiation, and increased expression of genes involved in immunoglobulin production and plasma cell function, including *PRDM1*, *CD27*, *MZB1*, and *XBP1* (Figure S2C). B cells also displayed IL-12 pathway enrichment and increased CXCR4 (Figure S2D),^34^ suggesting a potential adaptation to the altered placental microenvironment in PreE.^35^

#### Spatial Transcriptomics Identify Compartment-Specific Immune Signaling in PreE

Spatial profiling (10× Genomics, Xenium) on placental sections encompassing both maternal (decidua) and fetal (chorionic villi) compartments (identified by hematoxylin-eosin and human leukocyte antigen G positivity; Figure S3A) mapped all single-cell–defined cell types across the placenta, including multinucleated STBs (Figure S3B). PreE placentas showed spatially segregated IFNγ signaling, with *IFNGR1* enriched in the fetal villous compartment, whereas *IFNGR2* was restricted to maternal decidua (Figure 1D). These data highlight spatially compartmentalized immune activation in PreE.

#### Nonimmune Cells Exhibit Inflammatory and Fibrotic Reprogramming in PreE

Trophoblasts, stromal cells, and ECs showed increases in PreE gene signature scores (Figure 1E, top) and enriched receptor-ligand interactions (Figure 1F), linked with complement activation, inflammation, and fibrotic patterning (Figures S4A, S4B, and S5A). Nonimmune cells displayed dysregulation of HIF-1α, AP-1, FAK (focal adhesion kinase), and β1 integrin signaling (Figure S5B), concomitant with widespread increases in fibrosis-associated gene modules (Figure 1E, middle).

#### Hippo Pathway Dysregulation Emerges as a Major Nonimmune Signaling Abnormality in PreE

During examination of dysregulated pathways in nonimmune cells, Hippo signaling emerged as one of the most striking alterations (Figure 1E, bottom). Multiple cell types showed enriched Hippo pathway activity (Figure 1G), and receptor–ligand analysis revealed extensive Hippo-associated communication among trophoblasts, stromal cells, and immune cells, suggesting broad pathway engagement across the PreE placenta. Within the Hippo pathway, we observed selective activation of *TEAD* transcription factors and *VGLL* family coregulators, but not *YAP1*, in PreE trophoblasts (Figure 1H; Figure S6A and S6B). TEAD1/3 and VGLL3 were significantly upregulated in PreE (Figure 1K; Table S2), indicating preferential engagement of the TEAD–VGLL3 transciptional axis.

#### VGLL3 Is Significantly Upregulated in PreE Trophoblasts and Physically Interacts with TEAD1/3

The proportion of *VGLL3*-expressing cells among PreE trophoblasts was significantly increased with expansion of VGLL3^hi^ trophoblast populations, beyond the discrete decidua-adjacent niches seen in healthy placentas (Figure 1I, 1J, and 1M). This distribution paralleled *FLT1* expression patterning (Figure S6C). Coimmunoprecipitation combined with mass spectrometry confirmed physical interactions between VGLL3 and TEAD1/TEAD3 (Figure 1K; Table S2). These findings substantiated spatial sequencing data displaying coexpression and localization of VGLL3, TEAD1, and TEAD3 in STBs and EVTs, with higher colocalization scores and odds ratios seen in PreE (Figure 1L). The proximity ligation assay further demonstrated increased in situ VGLL3 and TEAD1 proximity in PreE placentas, supporting this in vivo interaction (Figure 1N).Together, these findings establish VGLL3 as an active Hippo pathway cofactor in PreE trophoblasts.

#### Replication of VGLL3 Dysregulation in an Independent PreE Cohort

Consistent with our scRNA-seq dataset, reanalysis of a large bulk RNA-seq cohort (n=84; GSE25906) revealed highly concordant expression changes. Overlapping upregulated genes included *IGFBP1*, a gene with polymorphisms previously associated with increased PreE risk^36^; *DDX20*, a regulator of microRNA biogenesis in trophoblasts^37^ (Figure S7A); and enrichment of MHC class II, SMAD, and TGF-β pathways (Figure S7B). Moreover, upstream regulatory analysis identified *VGLL3* as a predicted regulator of the PreE signature (Figure S7C), supporting a central mechanistic role.

#### VGLL3 and TEAD1 Develop Diverging Differentiation Trajectories in PreE Trophoblasts

**Figure 2.**
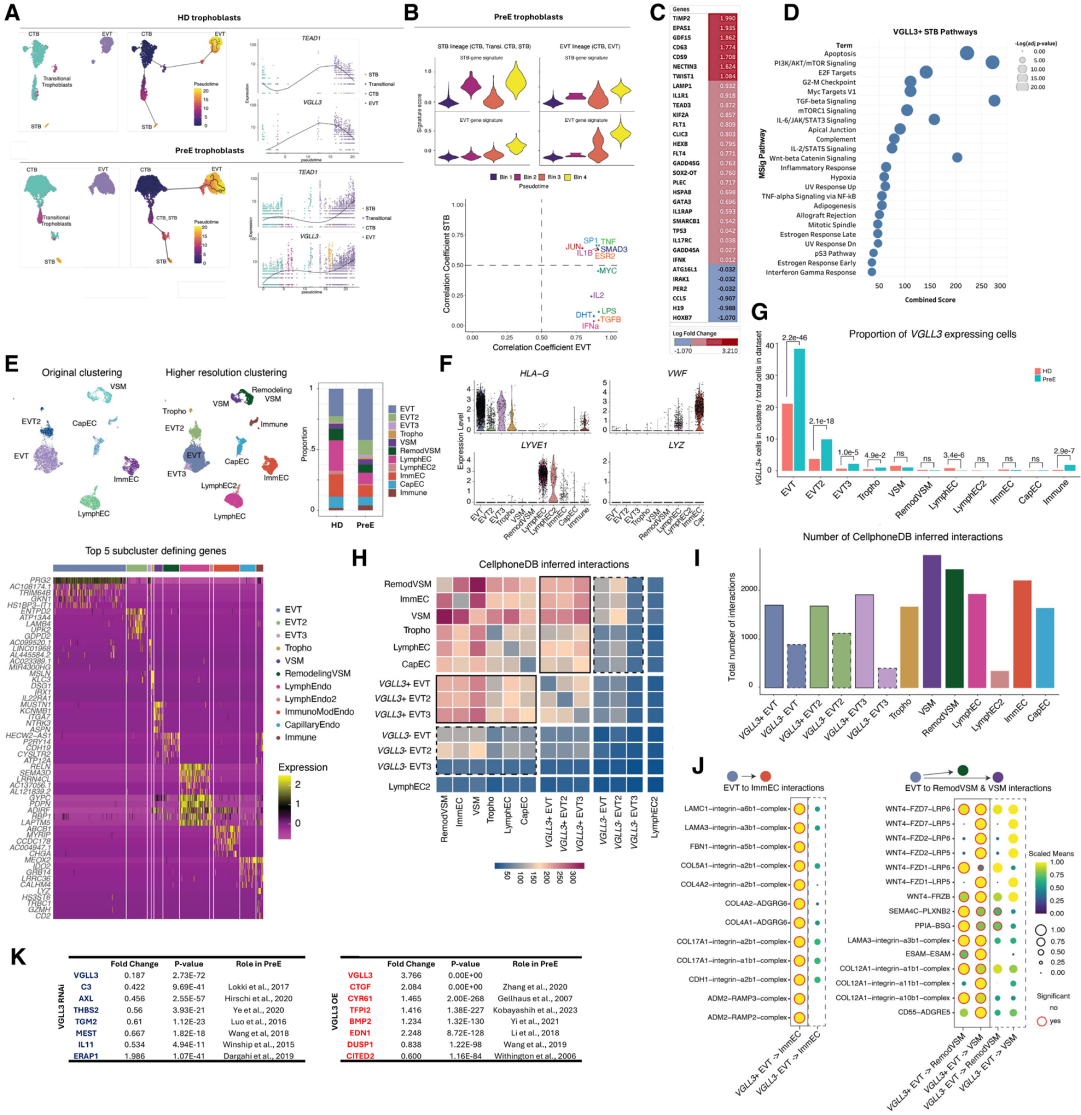
**Aberrant VGLL3 expression in preeclampsia (PreE) trophoblasts promotes inflammatory responses and apoptosis in syncytiotrophoblasts (STBs) and abnormal interaction of extravillous trophoblasts** (**EVTs) with immunomodulatory endothelial cells (ECs). A**, Trophoblast cells are shown on uniform manifold approximation and projection plots generated by Monocle3-derived dimensional reduction and colored coded by Seurat-defined clusters (**left**) or pseudotime value (**right**). Thick lines (**right**) are developmental trajectories defined by pseudotime analysis. Gene expression plots show expression of the indicated genes across pseudotime, color coded by Seurat-defined clusters. **B**, Violin plots show the module score of gene expression signatures across pseudotime bins (**top**). Signatures were defined by identifying genes that increased in trophoblast lineages across pseudotime that were also higher in PreE compared with healthy donors. Ingenuity Pathway Analysis was used to identify putative upstream regulators of these signatures. **Bottom**, Scatterplot showing the correlation coefficients between the module target score of putative upstream regulators and EVT pseudotime (*x* axis) and STB pseudotime (*y* axis). **C**, Select genes differentially expressed in VGLL3^+^ STB (*P*<0.05). **D**, Pathways dysregulated in VGLL3^+^ STBs vs VGLL3^–^ STBs based on the MSigDB Hallmark 2020 database. **E**, Uniform manifold approximation and projection plots showing EVT, endothelial, and vascular smooth muscle (VSM) cells from a human dataset, colored by original cluster (**top left**) or subcluster (**top middle**). The bar graph shows proportions of cells within conditions (**top right**). The heatmap lists cluster-defining genes (**bottom**). **F**, Violin plots showing expression of the indicated genes across subclusters. **G**, Bar plot showing the proportion of VGLL3^+^ cells in individual subclusters over total cells in the sample. Significance was calculated with a 2-sample test for equality of proportions with continuity correction, followed by Benjamini-Hochberg test for multiple comparisons. Adjusted *P* values are shown above each plot. **H**, CellphoneDB was used to identify inferred ligand-receptor interactions between VGLL3^+^ and VGLL3^−^ trophoblast populations and endothelial or VSM cells from preeclamptic placental tissue. The heatmap shows the number of ligand-receptor pairs between the indicated populations. A solid line indicates VGLL3^+^ cells, and a dashed line indicates VGLL3^−^ cells. **I**, Number of inferred interactions between the indicated populations. A solid line indicates VGLL3^+^ cells, and a dashed line indicates VGLL3^−^ cells. **J**,Dot plots showing scaled mean scores (the normalized means of the interaction strength between the indicated ligand and receptor) between VGLL3^+^ and VGLL3^−^ EVT and ImmEC cells (**left**) or EVT and VSM cells (**right**). **K**, Select PreE-associated DEGs after *VGLL3* knockdown (RNAi, **top**) in HTR8 cells or overexpression (OE, **bottom**) in HEK293 cells. CapEC indicates capillary endothelial cell; CTB, cytotrophoblast; ImmEC, immunomodulatory endothelial cell; LymphEC, lymphatic endothelial cell; Transi CTB, transitional cytotrophoblasts; Tropho, trophoblasts; VWF, von Willebrand Factor.

The Hippo signaling pathway has been implicated in regulating the stemness and differentiation of placental trophoblasts.^38^ To assess how TEAD1 and VGLL3 expression changes during trophoblast differentiation, we applied Monocle3 to our scRNA-seq data to infer developmental trajectories in trophoblast cells from both HD and PreE placentas.^39^ While the overall pseudotime trajectories were comparable between HD and PreE, the patterns for TEAD1 and VGLL3 expression developed differently, steadily increasing across pseudotime in PreE EVT cells versus the rise-and-fall pattern observed in HD samples (Figure 2A).

#### Inflammatory Upstream Regulators Drive PreE-Specific Transcriptional Programs in Differentiating Trophoblasts

To identify potential regulators of the PreE-specific gene expression changes during trophoblast differentiation, we defined gene signatures comprising transcripts that were upregulated across pseudotime in PreE EVTs or STBs and preferentially expressed in PreE over HD cells (Figure 2B, top). Next, Ingenuity Pathway Analysis was used to infer upstream regulators of these signatures. Next, for each predicted regulator, a module score was calculated on the basis of downstream target genes, and correlation with pseudotime was determined. This analysis identified several inflammatory drivers, including LPS, type I interferons, IL-2, and TNF, as candidate regulators of the PreE-associated transcriptional shift (Figure 2B, bottom). These findings demonstrate dysregulated trajectories of VGLL3 and TEAD1 during trophoblast differentiation that occur concomitant with a progressive, proinflammatory transcriptional program in PreE STBs and EVTs.

#### VGLL3⁺ STBs Show Activation of PreE-Associated Pathways

To further characterize functional differences between VGLL3^+^ and VGLL3^−^ STBs, we interrogated the spatial transcriptomics data and observed that VGLL3^+^ STBs contained increases in *TIMP2*,^40^ *GDF15*,^41^ and *FLT1*,^42^ genes previously implicated in PreE (Figure 2C). Pathway enrichment analysis showed activation of key signaling pathways, including apoptosis, PI3K/AKT/mTOR, TGF-β, and IL-6/JAK/STAT3 in VGLL3^+^ STBs (Figure 2D). These findings are highly relevant, given that STBs in PreE exhibit increased apoptosis, altered nutrient transport, and enhanced shedding of proinflammatory mediators, processes that contribute to the formation of syncytial knots and systemic maternal inflammation. These findings imply that VGLL3 acts upstream of these dysregulated processes, serving as a potential driver of STB dysfunction.

### VGLL3 Enhances EVT-Vascular Cell Interactions Linked to Fibrosis and Impaired Spiral Artery Remodeling

Successful pregnancy depends on the ability of EVTs to invade the maternal decidua and remodel spiral arteries, a process that involves the replacement of VSM cells and ECs. Impaired spiral artery remodeling is a hallmark of PreE. To better understand EVT interactions with ECs and VSMs, we performed high-resolution re-clustering of these populations and annotated subtypes using canonical markers. This included identification of endovascular EVTs, lymphatic endothelial cells, immunomodulatory ECs, and capillary ECs (Figure 2E and 2F). Across all EVT subtypes, PreE samples showed a significantly higher proportion of VGLL3^+^ cells compared with HD samples (Figure 2G). To assess whether VGLL3 expression influenced cell–cell communication, we conducted CellPhoneDB analysis^32^ and observed that VGLL3^+^ EVTs engage in more ligand-receptor interactions than VGLL3^−^ EVTs (Figure 2H and 2I). Notably, these enhanced interactions were concentrated between VGLL3^+^ EVTs and remodeling VSMs, mature VSMs, and immunomodulatory ECs (Figure 2H). Many of the VGLL3^+^-specific interactions were enriched for collagen-related ligands, fibrotic signaling molecules, and components of the WNT signaling pathway (Figure 2J), supporting a role of VGLL3 in promoting fibrosis and vascular dysfunction in PreE.

#### VGLL3 Defines a Coordinated Gene Network Driving Immune Activation and Pathogenic Signaling in PreE

To define the gene network centered around VGLL3 in trophoblasts, we analyzed VGLL3-correlated gene expression across 80 trophoblast microarray samples. To stratify 6634 genes with positive correlation with VGLL3 (Spearman’s ρ>0), we applied a graphical method identifying a critical inflection point in the correlation decay curve. This yielded a refined set of 737 genes with strong VGLL3 correlation (ρ≥0.67) that constituted a localized transcriptional subnetwork (Figure S7D, top). Among the top VGLL3-correlated genes were *PSG2*, *PSG3*, *TGFBR2*, and *CSF2RB* (ρ≥0.88) (Figure S7D, bottom). Functional enrichment analysis determined that this VGLL3-associated network was highly enriched for “granulocyte activation,” “cell activation involved in immune response,” “neutrophil degranulation,” and “myeloid leukocyte–mediated immunity” (Figure S7D, bottom). To directly assess the transcriptional impact of VGLL3, we performed bulk RNA-seq on HEK293 cells and HTR8 trophoblasts following VGLL3 overexpression or knockdown, respectively. Differential expression analysis identified several key PreE-associated genes, including *C3*, *AXL*, *CTGF*, and *CYR61*, among the top VGLL3-regulated targets (Figure 2K), underscoring the role of VGLL3 as a potential upstream regulator of pathogenic gene expression in PreE.^43–51^

### Placental Overexpression of Vgll3 Triggers a PreE-Like Phenotype in Pregnant Mice

**Figure 3.**
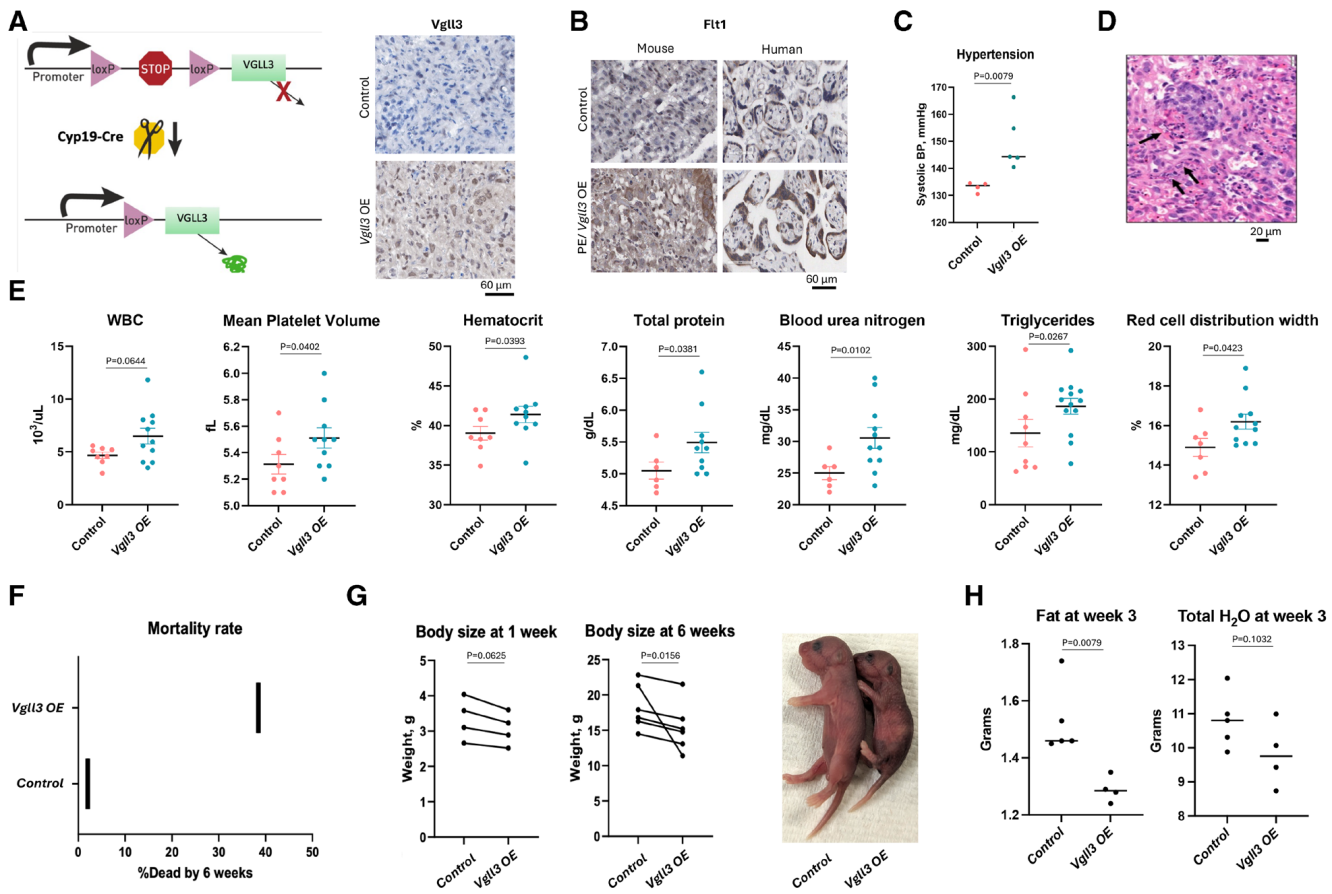
**Placental overexpression of *Vgll3* is sufficient to trigger a preeclampsia-like phenotype in pregnant mice. A**, Strategy for genetic engineering of mice with *Vgll3*-overexpressing (OE) placentas. Increased levels of Vgll3 were confirmed by immunohistochemistry (**right**). **B**, Upregulation of FLT1 was confirmed in human preeclampsia placentas and murine *Vgll3-OE* placentas by immunohistochemistry. **C**, Elevation in systolic blood pressure (BP) in pregnant dams with Vgll3-OE placentas determined by a tail cuff method on embryonic day 16.5. Significance was calculated with Mann-Whitney U test. **D**, Hematoxylin-eosin staining revealed microthrombi and fibrinoidnecrosis (arrows) around capillaries in the*Vgll3-OE* placentas. **E**, Circulatory white blood cells (WBC) and other blood parameters measured in late gestation (*P≤0.05). Significance was calculated with Mann-Whitney U test. **F**, Pups born from *Vgll3-OE* placentas exhibited increased mortality. **G**, Pups born from *Vgll3-OE* placentas exhibited smaller body size. Significance was calculated with paired *t* test. Normal distribution was determined by Wilcoxon matched-pairs signed rank test. Littermates born from the same dams but with different placental genotypes were matched for comparison. **H**, Body composition was measured at 3 weeks (*P≤0.05). Significance was calculated with Mann-Whitney U test. PreE indicates preeclampsia.

To determine whether placental overexpression of *Vgll3* is sufficient to induce PreE-like symptoms, we used a Cre/LoxP approach to overexpress *Vgll3* in placental trophoblasts in mice^52^ (Figure 3A). *Vgll3*-OE mouse placentas developed increases in *Flt1*, mirroring the phenotype observed in human PreE placentas (Figure 3B). Pregnant dams carrying *Vgll3*-OE placentas developed gestational hypertension (Figure 3C), accompanied by reduced cardiac ejection fraction (Figure S8A). Histological examination of placentas identified the presence of fibrinoid necrosis and microthrombus formation around placental capillaries (Figure 3D), along with immune cell infiltration (Figure S8B). Maternal blood profiling revealed elevated levels of mean platelet volume, hematocrit, total protein, blood urea nitrogen, triglycerides, and red blood cell distribution width (Figure 3E), while PlGF was significantly reduced (Figure S8C), a hallmark feature of human PreE. Offspring from *Vgll3*-OE mice had a 40% postnatal mortality rate (Figure 3F), reduced body weight (Figure 3G), and lower adiposity by 3 weeks (Figure 3H). These findings demonstrate that trophoblast-specific *Vgll3* overexpression is sufficient to induce PreE-like pathology and impair fetal growth.

**Figure 4.**
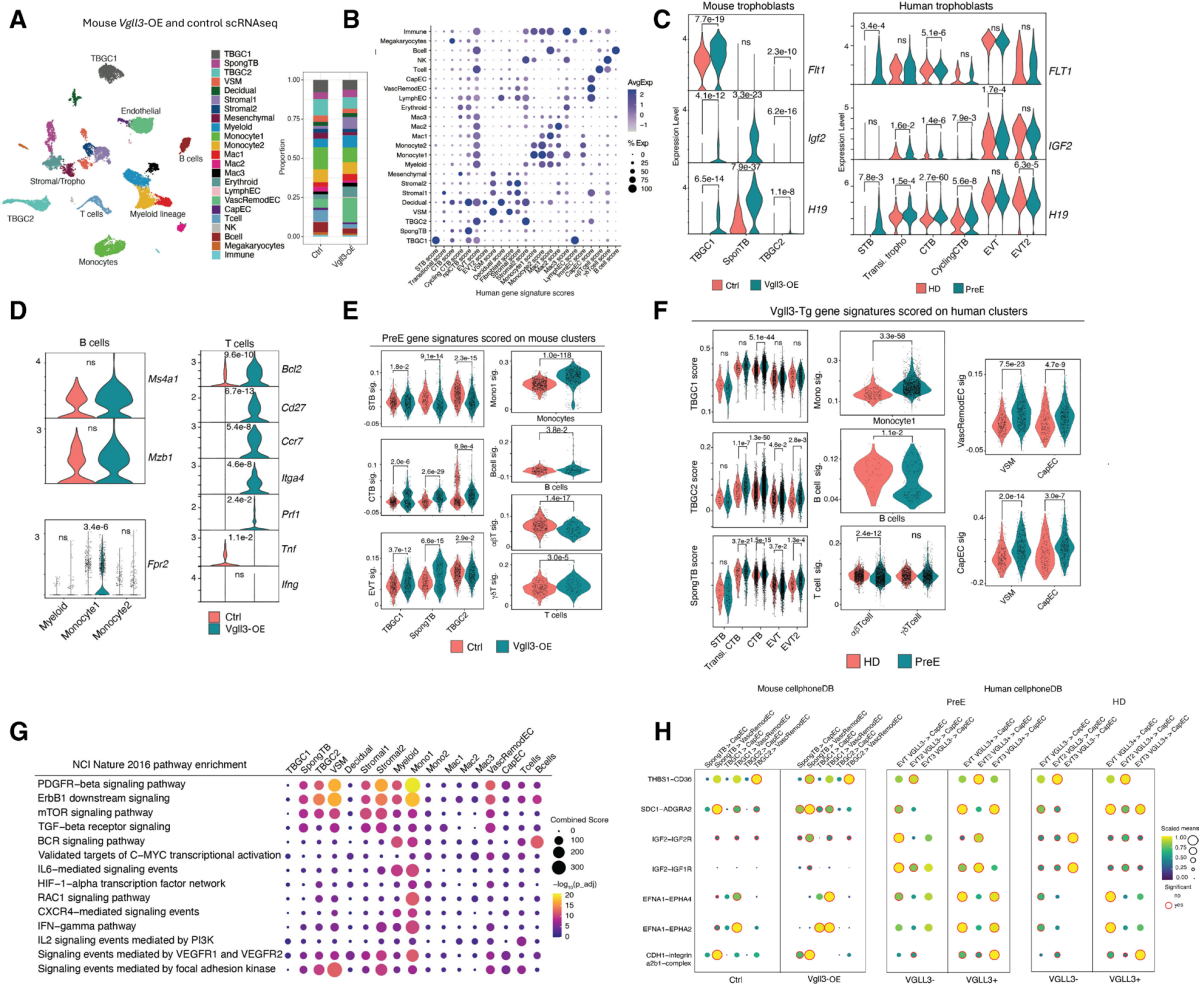
**Cells from Vgll3-overexpressing (OE) placentas display preeclampsia (PreE)–associated transcriptomic changes. A**, Uniform manifold approximation and projection plot showing mouse placental cells colored by subtypes (**left**) and proportions of clusters in each genotype (**right**). **B**, Dot plot of signature scores derived from human clusters in Figure 1A. **C**, Violin plots showing expression of the indicated genes in mouse trophoblasts and their corresponding orthologs in human trophoblasts. Significance was calculated with Wilcoxon rank sum test. *P* values are shown above the plots. **D** and **E**, Violin plots showing expression of indicated mouse genes (**D**) or module scores (**E**) in select clusters separated by genotype. Significance was calculated with Wilcoxon rank sum test followed by Bonferroni correction using all genes in the dataset (**D**) or Benjamini-Hochberg false discovery rate correction (**E**). Adjusted *P* values are shown above the plots. **F**, Violin plots showing module scores of human orthologs of *Vgll3-OE*–specific signatures on select human clusters. Significance was calculated with Wilcoxon rank sum test followed by Benjamini-Hochberg false discovery rate correction. Adjusted *P* values are shown above the plots. **G**, Double-axis scatterplot of pathways dysregulated in *Vgll3-OE* placentas based on the NCI-Nature 2016 database. **H**, Dot plots showing scaled mean scores of indicated interactions between trophoblast lineage cells and endothelial cells in mice (**left**) and human (**right**). CapEC indicates capillary endothelial cell; CTB, cytotrophoblast; Ctrl, control; EVT, extravillous trophoblast; HD, healthy donor; LymphEC, lymphatic endothelial cell; Mac, macrophages; NK, natural killer cells; ns, not significant; scRNA-seq, single-cell RNA sequencing; sig, signature; SpongTB, spongiotrophoblast; STB, syncytiotrophoblast; Tropho, trophoblasts; VascRemodEC, vascular remodeling endothelial cells; and VSM, vascular smooth muscle.

To evaluate the impact of *Vgll3* overexpression on the placental transcriptome, we performed scRNA-seq on control and *Vgll3*-OE mouse placentas (Figure 4A). Cell clusters were annotated using both mouse-specific marker genes and human signature scores (Figure 4B). While the mouse placenta is different cellularly than the human placenta, the trophoblast compartment includes trophoblast giant cells and spongiotrophoblasts. *Vgll3*-OE placental trophoblasts show elevated expression of *Flt1*, *Igf2*, and *H19*, mirroring human PreE trophoblasts (Figure 4C), along with increased activation of the mTOR signaling pathway (Figure S8D and S8E). B cells, myeloid cells, and T cells also exhibited transcriptional changes consistent with human PreE (Figure 4D).

To assess PreE signature enrichment, we identified PreE-specific genes (log_2_FC [fold change] >0.1, *P*<0.05) from human EVT, STB, and CTB clusters and scored their mouse orthologs across mouse cell types (Figure 4E; Table S3). Trophoblasts and monocytes from *Vgll3*-OE placentas displayed significant upregulation of these PreE signatures (Figure 4F). A reciprocal analysis using a *Vgll3*-OE mouse signature further confirmed elevated scores in human PreE CTB, EVT, and monocyte clusters compared to HD samples (Figure 4E). These findings indicate that *Vgll3* drives conserved PreE-associated transcriptional programs in both trophoblasts and monocytes across species. Pathway analysis of *Vgll3*-OE placentas identified PDGFR-β, ErbB1, and mTOR signaling as top dysregulated pathways in these cell types (Figure 4G).

Last, we analyzed ligand-receptor interactions in the mouse dataset and found that trophoblast–capillary EC interactions involving receptors critical for vascular integrity and angiogenesis were significantly enriched in dams with *Vgll3*-OE placentas compared with controls (Figure 4H). These same interactions were also elevated in human VGLL3^+^ EVTs relative to VGLL3^−^ EVTs (Figure 4H). Together, these findings demonstrate that *Vgll3* overexpression in placentas induces PreE-like phenotypes and provides a viable in vivo model to study cellular and molecular mechanisms underlying PreE.

### Deletion of Vgll3 Suppresses Placental Inflammation in Mice but Does Not Affect Pregnancy Progression

**Figure 5.**
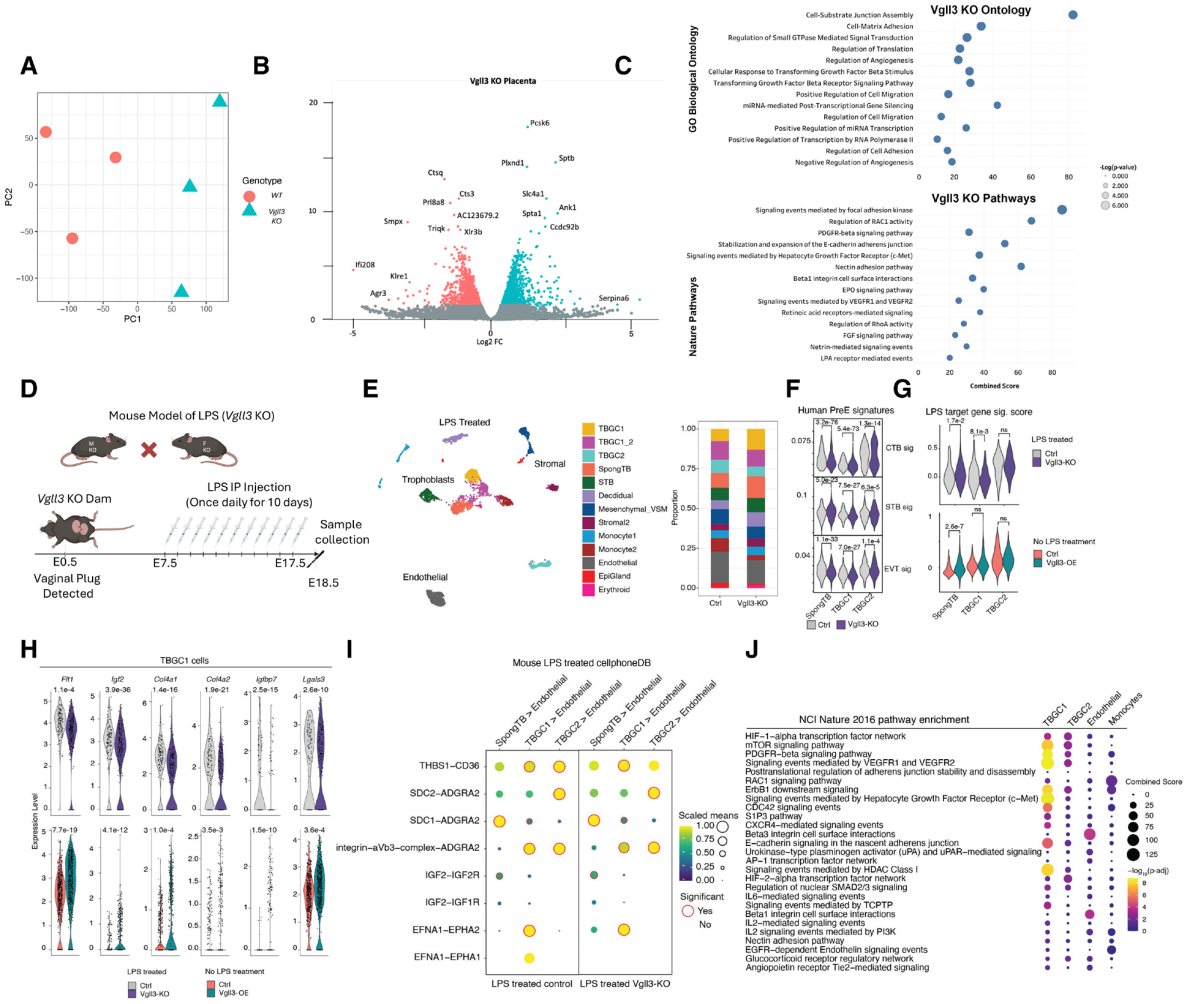
**Deletion of *Vgll3* suppresses placental inflammation in mice. A**, Principal component (PC) scatterplot showing distinct separation of placenta transcriptomes from murine wild-type (WT) and *Vgll3 knockout* (KO) mice. **B**, Volcano plot with top DEGs in *Vgll3* KO placentas (n=3 per group). **C**,Bar graphs with top dysregulated pathways in *Vgll3* KO placentas according to the GO Biological Process 2023 and NCI-Nature 2016 databases. **D**,Mouse model of placental inflammation in the absence of *Vgll3*. Data of lipopolysaccharide (LPS)–treated placenta shown in **E** through **J** are representative of 2 independent experiments. Statistics were calculated on a pooled dataset. **E**, Uniform manifold approximation and projection plot showing cells from placenta of LPS-treated WT and *Vgll3* KO mice and their proportions. **F**, Human preeclampsia (PreE)–specific gene expression module within major trophoblast populations scored across murine trophoblasts from WT (n=2) or *Vgll3* KO (n=3) mice treated with LPS. Significance was calculated with Wilcoxon rank sum test followed by Benjamini-Hochberg false discovery rate correction. Adjusted *P* values are shown above the plot. **G**, Module score of LPS-targeted genes calculated in murine placenta trophoblasts from either (**top**) WT or *Vgll3* KO mice treated with LPS or (**bottom**) untreated WT or *Vgll3*-overexpressing (OE) mice. Significance was calculated with Wilcoxon rank sum test followed by Benjamini-Hochberg false discovery rate correction. Adjusted *P* values are shown above the plot. **H**, Violin plots showing expression of the indicated PreE-associated genes in TBGC1 trophoblasts. Significance was calculated with Wilcoxon rank sum test. *P* values are shown above the plots. **I**, Dot plots showing scaled mean scores of the indicated interactions between trophoblast-lineage cells and endothelial cells in LPS-treated mice. **J**, Enriched pathways (Panther 2016 database) for genes downregulated in LPS-treated *Vgll3* KO vs WT cells. Each pathway is plotted by its −log_10_(adjusted *P* value), with bubble size indicating the combined score. E indicates embryonic day; CTB, cytotrophoblast; Ctrl, control; EVT, extravillous trophoblast; FC, fold change; GO, Gene Ontology; IP, intraperitoneal; sig, signature; SpongTB, spongiotrophoblast; STB, syncytiotrophoblast; and VSM, vascular smooth muscle.

To assess whether VGLL3 is essential for normal pregnancy, we monitored global *Vgll3* KO mice throughout gestation. *Vgll3* KO dams showed no overt abnormalities, and their pregnancies and offspring were comparable with wild-type (WT) controls. Transcriptomic profiling of KO placentas revealed distinct gene expression clustering (Figure 5A). Among the most upregulated genes in *Vgll3* KO placentas was *Pcsk6* (Figure 5B), a gene previously implicated in blood pressure regulation in PreE, with *Pcsk6* deficiency linked to salt-sensitive hypertension.^53^ Enrichment analysis of upregulated genes pointed to pathways involved in “positive regulation of cell migration” and “regulation of angiogenesis,” whereas downregulated genes were associated with “signaling events mediated by VEGFR1 and VEGFR2” (Figure 5C).

Our analyses identified LPS as a potential upstream regulator of the PreE-specific transcriptomic shift in human placentas (Figure 1B), consistent with previous studies in which LPS was used to induce PreE-like inflammation in animal models.^54^ To assess whether *Vgll3* deletion mitigates LPS-induced inflammation, we administered daily intraperitoneal LPS injections (Sigma-Aldrich, L2630, 20 μg/kg) to pregnant WT and *Vgll3* KO dams from E7.5 to E17.5 (Figure 5D) and performed scRNA-seq on placentas collected on day 18.5 (Figure 5E, left). Deletion of *Vgll3* (in the presence of LPS) did not substantially alter the composition of major placental cell clusters (Figure 5E, right). However, scoring using a human PreE-specific gene signature revealed that TBGC1 cells from *Vgll3* KO mice showed reduced expression of STB, CTB, and EVT PreE gene signatures (Figure 5F). Moreover, *Vgll3* KO trophoblasts displayed decreased expression of an LPS-induced gene signature (Figure 5G, top). In contrast, trophoblasts from *Vgll3*-OE placentas (in the absence of LPS) displayed increased expression of this same LPS signature compared with controls (Figure 5G, bottom). These transcriptional changes were accompanied by a modest, though not statistically significant, reduction in blood pressure in LPS-treated *Vgll3* KO dams compared with LPS-treated WT controls (Figure S9).

#### VGLL3 Is Required for FLT1 Regulation and Pathologic Trophoblast–Endothelial Cell Communication in PreE

Supporting a role of *Vgll3* in regulating *Flt1* and adhesion and extracellular matrix remodeling, we observed reduced expression of *Flt1*, *Igf2*, *Col4a1*, *COl4a2*, *Igfbp7*, and *Lgals3* in trophoblasts from LPS-injected *Vgll3* KO dams compared with LPS-injected WT controls, and conversely, increased expression in *Vgll3*-OE placentas compared with WT controls (Figure 5H). Ligand-receptor interaction analysis revealed that PreE-specific trophoblast-EC interactions identified in the *Vgll3*-OE mouse model were significantly enriched in LPS-treated WT mice but diminished in LPS-treated *Vgll3* KO mice (Figure 5I). Among the top differentially regulated pathways in LPS-treated *Vgll3* KO trophoblasts were HIF-1α and angiogenesis signaling (Figure 5J). These findings underscore the importance of *Vgll3* in driving inflammatory, extracellular matrix remodeling, and adhesive signaling in trophoblasts and its broader impact on placental cell–cell communication. Given the effectiveness of *Vgll3* deletion in attenuating placental inflammation in mice, we next sought to target VGLL3/Hippo signaling in human PreE placentas.

### Interference With Hippo Signaling Suppresses Disease Signaling in PreE Placentas ex Vivo

While a specific VGLL3 inhibitor is not yet available, verteporfin is a known inhibitor of the Hippo pathway at the transcriptional level.^55^ To evaluate whether verteporfin mimics the effects of VGLL3 inhibition, we treated HTR8 trophoblasts with verteporfin or VGLL3-targeting siRNA and performed bulk RNA-seq. Both treatments impacted overlapping pathways, including integrin-mediated cell surface interactions, angiogenesis, p53 effector signaling, and Syndecan-1 signaling (Figure S10A).

Comparison of DEGs revealed substantial overlap: 1191 of the 1493 DEGs identified in VGLL3 siRNA–treated cells (80%) were also differentially expressed in verteporfin-treated cells (Figure S10B). Notably, verteporfin induced broader transcriptomic changes than VGLL3 siRNA, likely reflecting additional VGLL3-independent effects. We categorized DEGs into those shared between treatments and those unique to each condition (Figure S10C) and performed pathway enrichment analyses for each group (Figure S10D). The overlapping DEGs were enriched in pathways associated with apoptosis, TNF signaling, hypoxia, and mTORC signaling, all previously implicated in human PreE and the *Vgll3*-OE mouse model.

**Figure 6.**
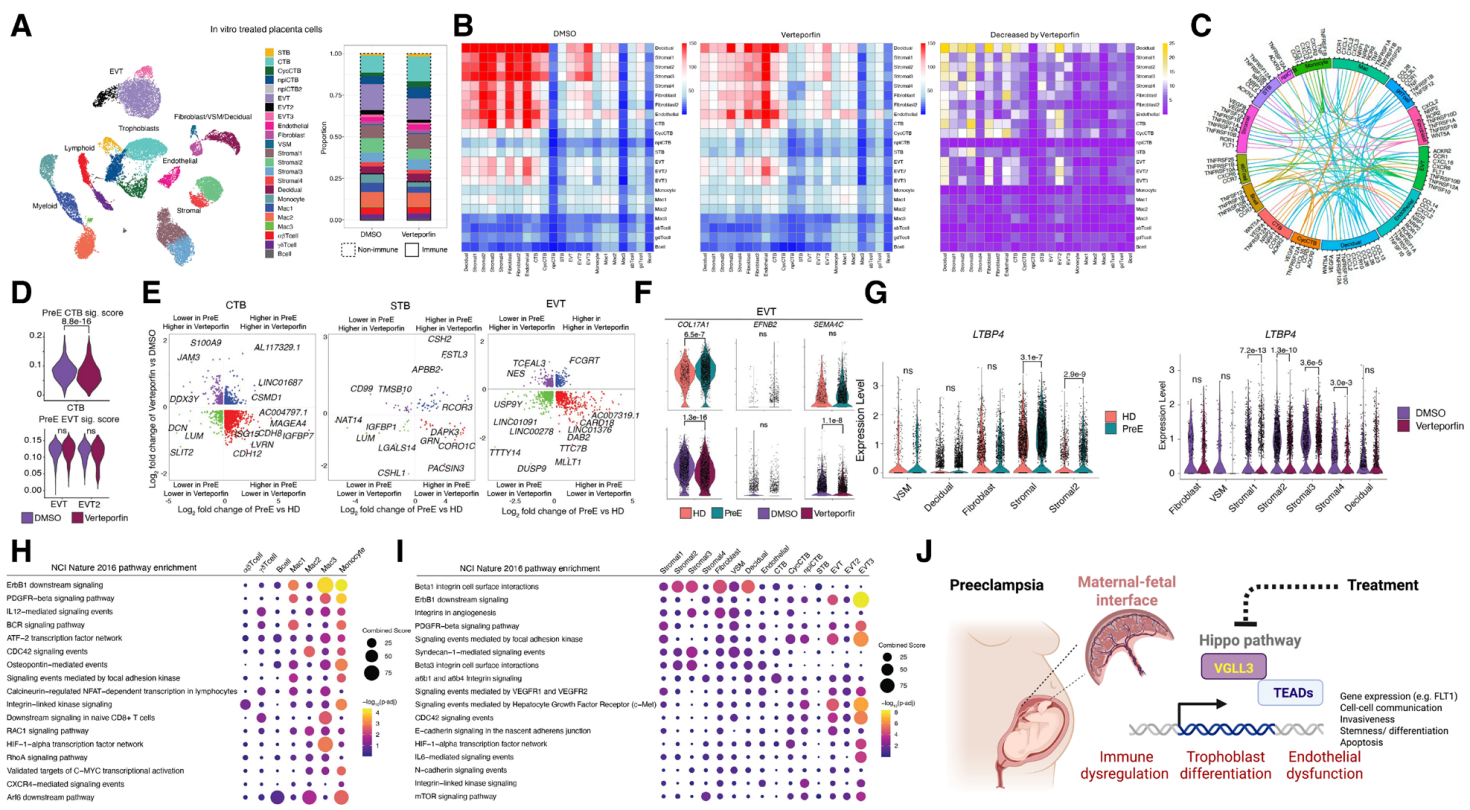
**Targeting the Hippo pathway suppresses preeclampsia (PreE) disease signaling ex vivo. A**, Uniform manifold approximation and projection plot showing human placental cells in placentas obtained from patients with PreE and treated with verteporfin or DMSO ex vivo, colored by cluster (**left**), with a bar graph showing proportions of clusters in each treatment (**right**). **B**, Heatmaps showing the number of ligand-receptor pairs between the indicated populations in the 2 treatment groups (**left** and **middle**) and as reduced by verteporfin (**right**). **C**,Circos plot depicting cytokine interactions reduced by verteporfin. **D**, Violin plots showing module scores of PreE cytotrophoblast (CTB) (**top**) or extravillous trophoblast (EVT) (**bottom**) gene signatures on the indicated cell clusters in DMSO- or verteporfin-treated placentas. Significance was calculated with Wilcoxon rank sum test followed by Benjamini-Hochberg false discovery rate correction. Adjusted *P* values are shown above the plots. **E**, Scatterplots showing PreE/healthy donor (HD) differential gene expression in the indicated CTBs, syncytiotrophoblast (STBs), or EVTs from untreated samples shown in Figure 1A (log2 fold change, *x* axis) vs verteporfin/DMSO differential gene expression (log2 fold change, *y* axis). Red dots indicate cells that are higher in PreE trophoblasts and repressed in verteporfin-treated cells. **F**, Violin plots showing expression of the indicated genes in EVTs from PreE and HD untreated placentas (**top**) and in ex vivo–treated PreE placentas (**bottom**). Significance was calculated with Wilcoxon rank sum test. Adjusted *P* values are shown above the plots. **G**, Violin plots of *LTBP4*, a gene targeted by VGLL3, in PreE placentas (vs HD controls) and in verteporfin-treated PreE placentas (vs DMSO-treated controls). Significance was calculated with Wilcoxon rank sum test followed by Bonferroni correction using all genes in the dataset. Adjusted *P* values are shown above the plots. **H** and **I**, Double-axis scatterplot of pathways differentially regulated by verteporfin in PreE placentas in immune (**H**) and nonimmune (**I**) cells on the basis of the NCI-Nature 2016 database. **J**, Schematic of VGLL3-driven dysregulation at the maternal-fetal interface in PreE. CycCTB indicates cycling cytotrophoblasts; Mac, macrophages; npiCTB, non-proliferative interstitial cytotrophoblasts; ns, not significant; sig, signature; and VSM, vascular smooth muscle. Figure 6J was created in BioRender. Gudjonsson, J. (2026) https://BioRender.com/s78z875.

Next, PreE placentas were treated ex vivo with verteporfin, followed by scRNA-seq analysis (Figure 6A, left). Verteporfin treatment did not induce major shifts in overall cellular composition compared with vehicle controls (Figure 6A, right). However, receptor–ligand expression analysis demonstrated that verteporfin broadly suppressed PreE-associated intercellular communication, most notably among decidual cells, stromal cells, ECs, and trophoblasts (Figure 6B).

Correspondingly, cytokine signaling was reduced across all major cell types with verteporfin treatment (Figure 6C). Verteporfin treatment also attenuated PreE gene signature scores, previously derived from comparisons between untreated PreE and HD placentas, in both CTBs and EVTs (Figure 6D). To further dissect verteporfin’s transcriptional effects, we compared DEGs from disease-state (PreE versus HD) and treatment (verteporfin versus vehicle control) in trophoblasts (Figure 6E). Multiple genes elevated in PreE were downregulated by verteporfin, including *IGFBP7*, *COL17A1*, *EFNB1*, and *SEMA4C* (Figure 6E and 6F). In stromal cells, *LTBP4*, a regulator of TGF-β activity and a recently identified VGLL3 target in salmon sexual maturation,^56–58^ was significantly elevated in PreE compared with HD and downregulated following verteporfin treatment (Figure 6G).

Pathway enrichment analysis of verteporfin-affected genes in immune cells highlighted suppression of ERBB1, IL-12, and BCR signaling (Figure 6H), while in nonimmune cells, verteporfin perturbed pathways involving integrin-mediated cell-surface interactions, angiogenesis, and FAK signaling (Figure 6I). Notably, verteporfin-treated placental explants exhibited a substantial overlap in DEGs and pathways with those affected by WRW4, an FPR2 antagonist (Figure S10E and S10F), consistent with prior findings that suppression of FPR2 mitigates inflammation in PreE.^59^ Verteporfin also decreased expression of multiple senescence-associated genes, including *BHLHE40* and *NDRG1*, that were aberrantly upregulated in PreE cytrophoblasts (Figure S11A). Some of these genes were also lower in the trophoblasts from *Vgll3* KO mice as compared with WT controls injected with LPS (Figure S11B), indicating that senescence in placental trophoblasts is a feature of PreE that is in part regulated by the Hippo pathway/VGLL3.

Taken together, these findings suggest that the dysregulation of the Hippo pathway in PreE leads to an immune imbalance at the maternal–fetal interface, alters trophoblast differentiation, and causes vascular endothelial dysfunction (Figure 6J). As these key pathological processes of PreE converge on a VGLL3-centered gene network, our data position VGLL3 as a pivotal master regulator in the etiology of PreE.

## DISCUSSION

It has been hypothesized that the female immune system evolved specialized adaptations to tolerate the immunologically invasive placenta while maintaining heightened immune surveillance against pathogens. While this balance may confer protection against infections and cancer, it also introduces female-specific immune signaling that increases susceptibility to autoimmune diseases. In the context of pregnancy, this immune complexity may contribute to dysregulated maternal–fetal immune interactions, predisposing some women to PreE.^60^

Pregnancy is a unique immunological state in which trophoblast invasion and fetal development depend on a finely tuned local inflammatory environment that supports cell clearance, angiogenesis, cell growth, and immune tolerance,^61^ processes that are disrupted in PreE.^2^ The Hippo signaling pathway, an evolutionarily conserved regulator of cell proliferation, differentiation, apoptosis, and mechanotransduction,^62^ has been implicated in fibrosis and endothelial dysfunction in autoimmune diseases.^63^ Emerging evidence suggests that, in pregnancy, the Hippo pathway regulates trophoblast stemness,^38,64^ angiogenesis,^65^ and maternal–fetal immune tolerance^66^ and may contribute to pregnancy complications,^67^ including PreE.^68,69^ Through detailed transcriptomic analyses of immune and nonimmune cells in PreE placentas, we identify dysregulation of the Hippo pathway as a central feature of disease pathogenesis, with the transcriptional coregulator VGLL3 emerging as a key mediator linking Hippo signaling to immune activation, trophoblast dysfunction, and endothelial pathology in PreE.

Our group previously identified VGLL3 as a regulator of a proinflammatory gene network in the skin that predisposes women to autoimmune diseases,^25^ including cutaneous lupus and Sjögren’s syndrome, both of which show VGLL3 upregulation. Notably, epidermal overexpression of *Vgll3* in mice is sufficient to induce a lupus-like autoimmune phenotype, characterized by hallmark features such as autoantibody production.^26^ Beyond autoimmunity, VGLL3 has emerged as a key regulator at the intersection of sex-specific biology and development. Independent studies have linked VGLL3 to the timing of sexual maturity in Atlantic salmon^57,58,70,71^ and to age of menarche in humans,^72^ an important clinical connection, as early menarche is associated with increased risk of both PreE^8,9^ and autoimmune disease.^73^

Strikingly, VGLL3 expression is highest in the placenta compared with all other organs. VGLL3 exerts its transcriptional effects by binding TEAD transcription factors, activating Hippo pathway signaling, a mechanism best characterized in cancer.^74,75^ During pregnancy, trophoblast proliferation, differentiation, migration, and invasion of the maternal uterus and vasculature mirror features of malignant tumors, a phenomenon termed “trophoblast pseudotumorigenesis.”^76^ Our data suggest that VGLL3 may play a physiological role in placental development through its regulation of these processes. However, in PreE, VGLL3 appears to be dysregulated; its overexpression and interaction with TEAD1 and TEAD3 drive immune dysregulation and trophoblast dysfunction, ultimately contributing to vascular pathology and pregnancy-induced hypertension.

In summary, our findings identify VGLL3 as a central molecular link connecting PreE, sexual maturation, and autoimmunity, offering a mechanistic basis for long-recognized clinical associations between PE among these conditions. Important questions remain, including the upstream drivers of VGLL3 dysregulation in PreE. The specific contributions of TEAD1 and TEAD3 and racial differences in VGLL3 signaling also remain to be defined, representing key avenues for future investigation. Despite these limitations, our results highlight VGLL3 and its associated signaling network as promising therapeutic targets in PreE.

## ARTICLE INFORMATION

### Acknowledgments

The authors would like to thank Drs Guðrún Valdimarsdóttir, Nándor Gábor Than, and Smarajit Mondal for helpful scientific discussions; Drs Enze Xing, Xianying Xing, Vincent Van Drongelen, Mehrnaz Gharaee-Kermani, Ranjitha Uppala, and Nitin Kumar for assistance; Dr Gustavo Leone for generously sharing Cyp19-Cre mice (52); Steven Whitesall for help with BP monitoring; and Jennifer Fox for help with sequencing submissions. The authors would also like to acknowledge University of Michigan Advanced Genomics Core, Epigenomics Core, In Vivo Animal Core, Proteomics Resource Facility, Physiology Phenotyping Core, Animal Metabolic, Physiological Behavioral Phenotyping Core, Unit for Laboratory Animal Medicine, Skin Research Center, and Frankel Cardiovascular Center’s Michigan Biological Research Initiative on Sex Differences in Cardiovascular Disease (M-BRISC). This work was supported by the Taubman Medical Research Institute, NIH-P30-AR075043 (to Drs Gudjonsson, Dlugosz, Sarkar, and Tsoi), NIH-R01-AI183620 (to Dr Gudjonsson), K24AR076975 (to Dr Kahlenberg), K08-AR078251 (to Dr Billi), and an M-BRISC pilot grant (to Dr Plazyo).

### Author Contributions

Drs Plazyo and Gudjonsson conceived experiments, wrote the manuscript, and secured funding. Drs Plazyo, Peela, Young, Syu, Erba, Kirma, Chopp, Zhang, Bogle, Swindell, and Tsoi performed experiments, including data analyses. Drs Hesson and Langen provided human samples. Drs Bergin, Sarkar, Ward, Singer, Gallagher, Kahlenberg, Billi, Dlugosz, Ganesh, Tsoi, and Gudjonsson provided expertise and feedback.

### Disclosures

Dr Kahlenberg has received grant support from Q32 Bio, Celgene/Bristol-Myers Squibb, Ventus Therapeutics, Rome Therapeutics, and Janssen. Dr Kahlenberg has served on advisory boards for AstraZeneca, Biogen, Bristol-Myers Squibb, Eli Lilly, EMD Serrano, Exo Therapeutics, Gilead, GlaxoSmithKline, Aurinia Pharmaceuticals, Rome Therapeutics, Synthekine, Vivideon, and Ventus Therapeutics. Dr Gudjonsson has served on advisory boards for Bristol-Myers Squibb, Eli Lilly, AbbVie, Almirall, Novartis, Sanofi, Boehringer Ingelheim, and Incyte. Drs Gudjonsson and Plazyo have a patent pending on therapeutic targeting of VGLL3 in PreE, acquired by PreEmpt Bio.

### Supplemental Material

Figures S1–S11

Tables S1–S6

## Supplementary Material
