# Supplementary material for: Defective Trophoblast Differentiation, Endothelial Dysfunction, and Immune Dysregulation in Preeclampsia Coalesce on a Placental VGLL3-Centered Gene Network
Source: Circulation. 2026 Apr 9;153(22):1743–60. doi: 10.1161/CIRCULATIONAHA.125.076218 (PMC13120743; doi:10.1161/CIRCULATIONAHA.125.076218)
Supplement: Supplementary file 2 [file cir-153-1743-s002.pdf]

SUPPLEMENTAL MATERIALS

Supplemental Figure 1.

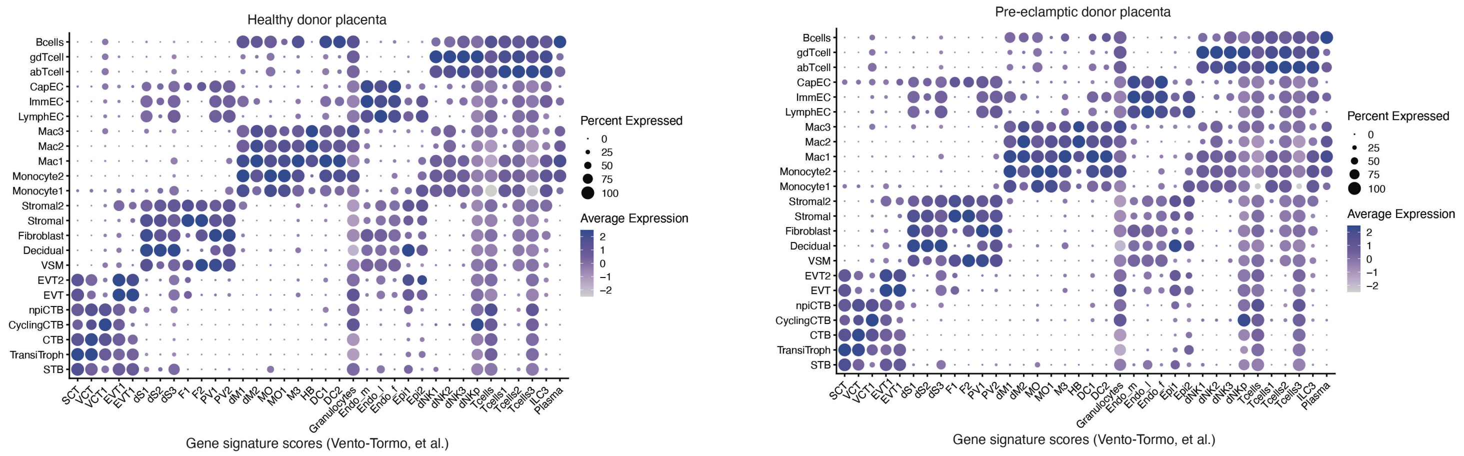

Supplemental Figure 2.

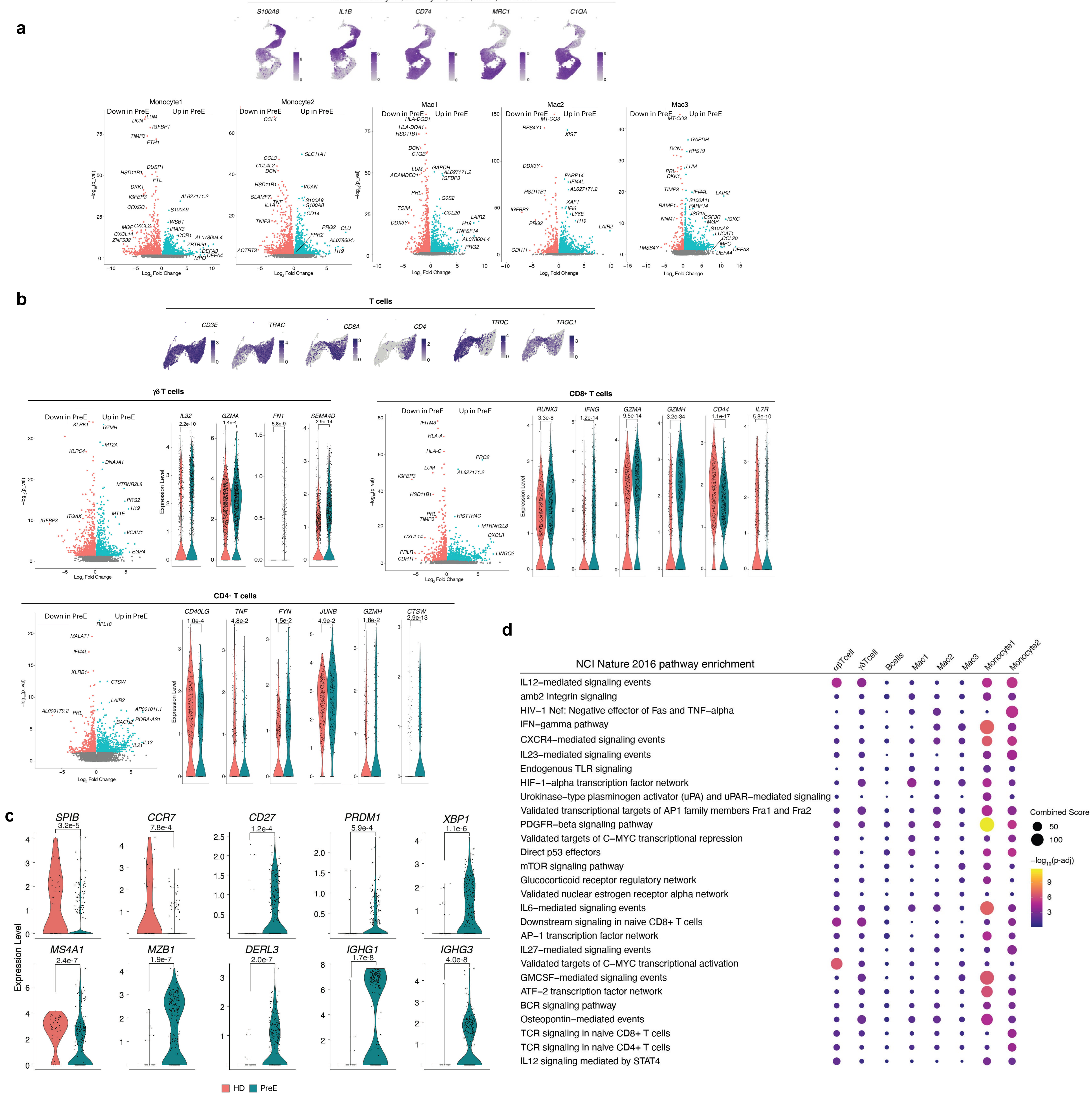

Supplemental Figure 3.

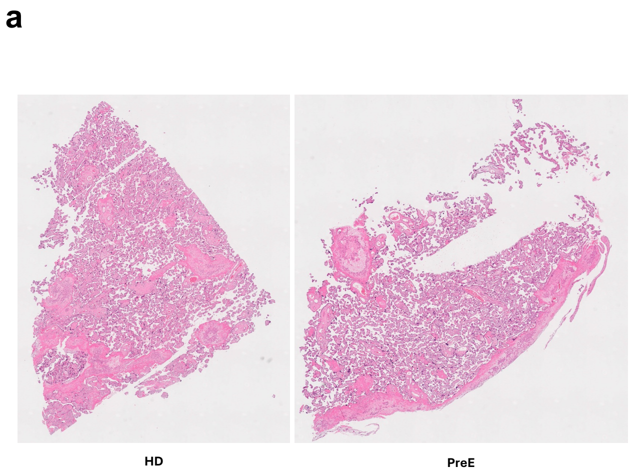

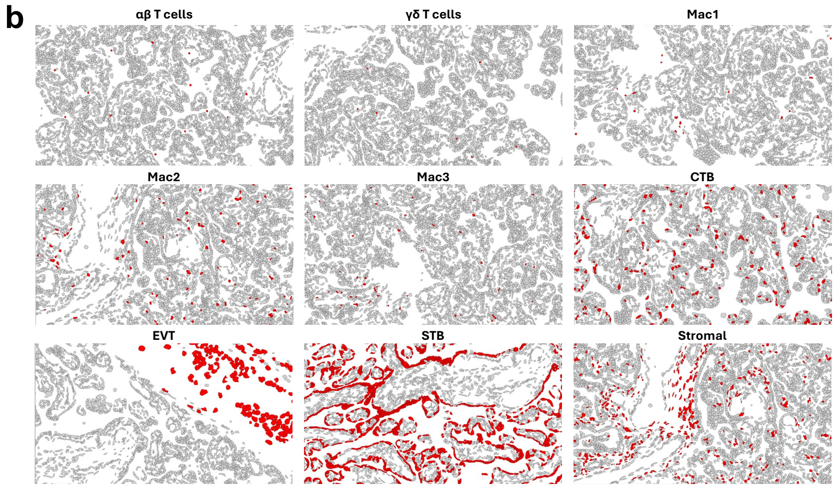

Supplemental Figure 4.

a

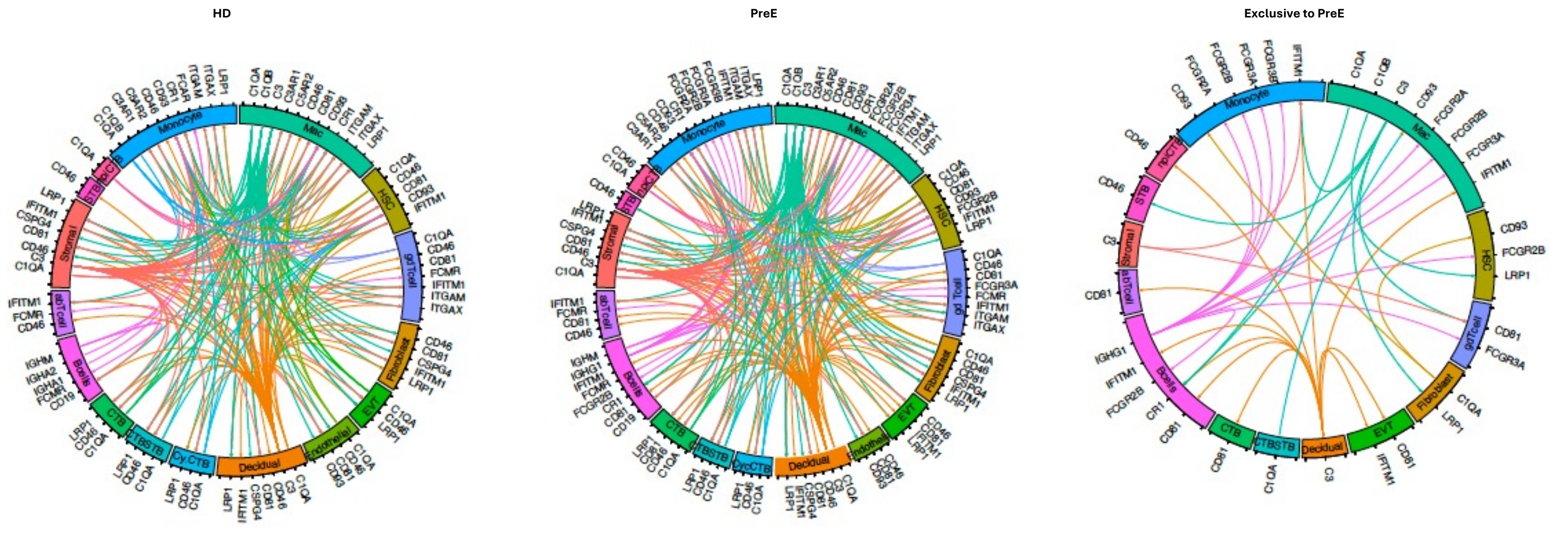

b

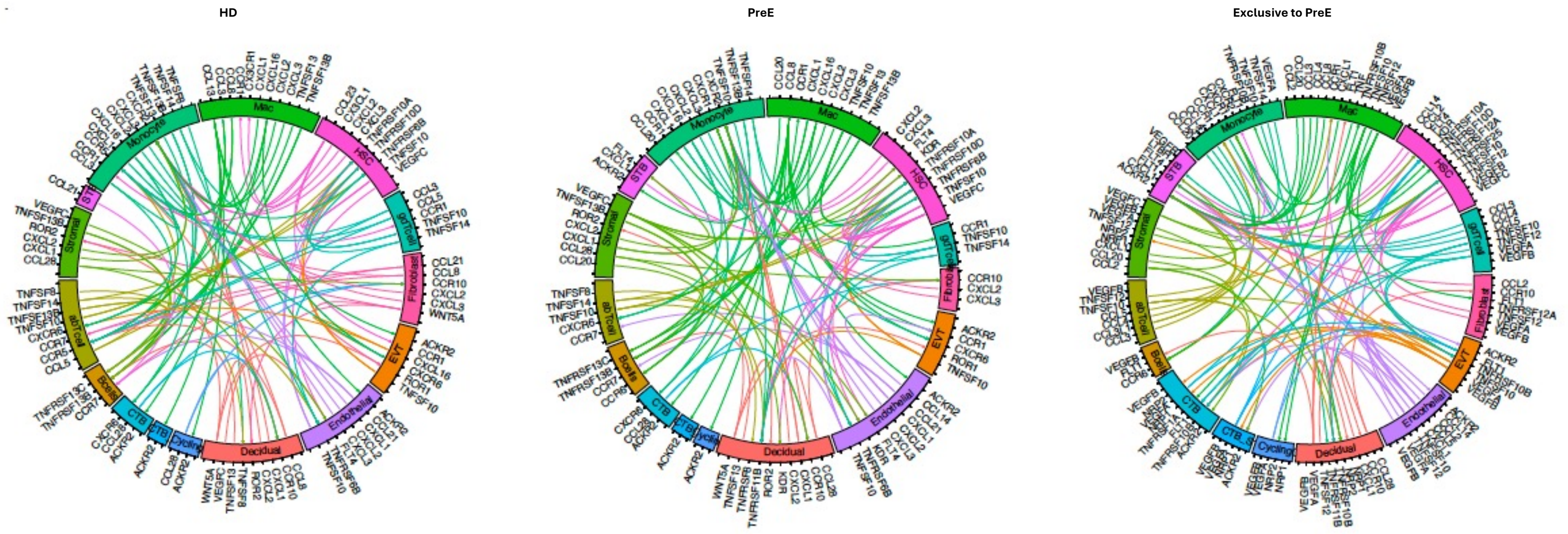

Supplemental Figure 5.

a b

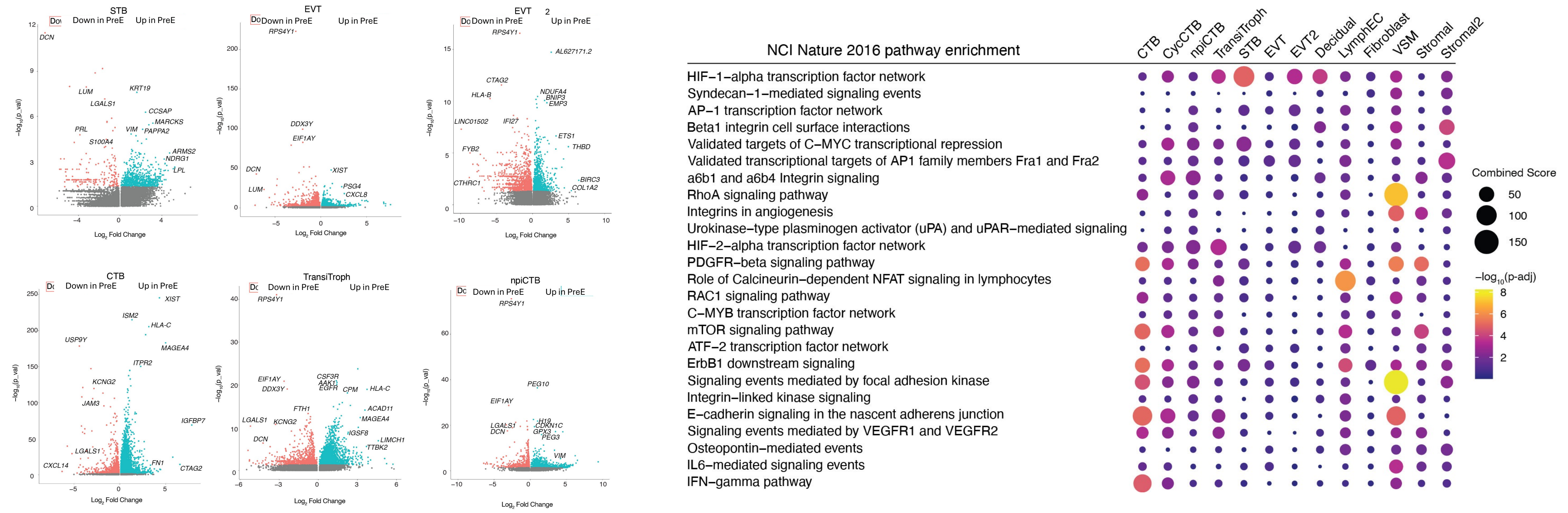

Supplemental Figure 6.

a

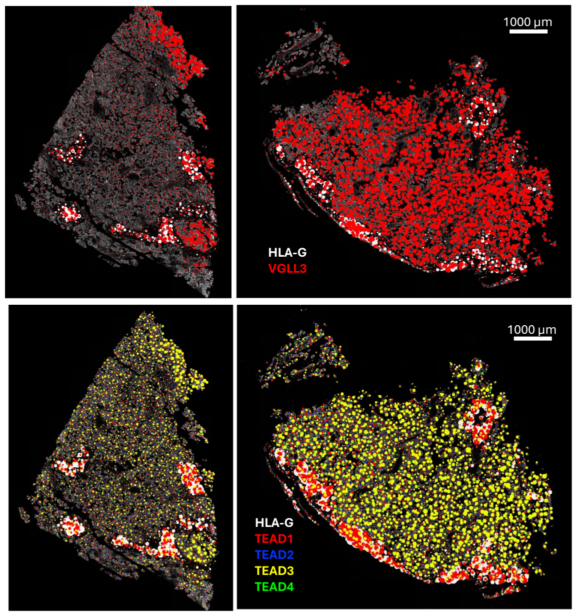

b

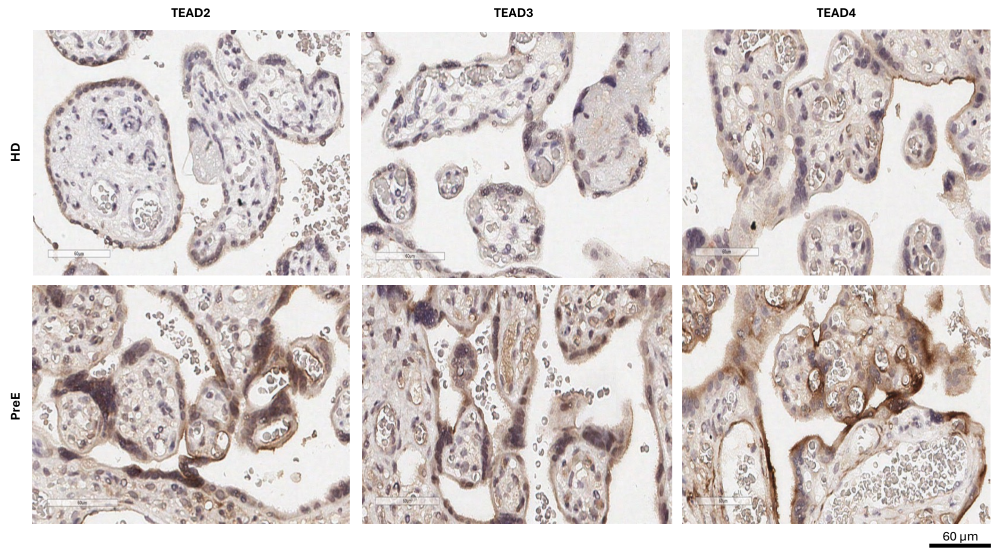

c

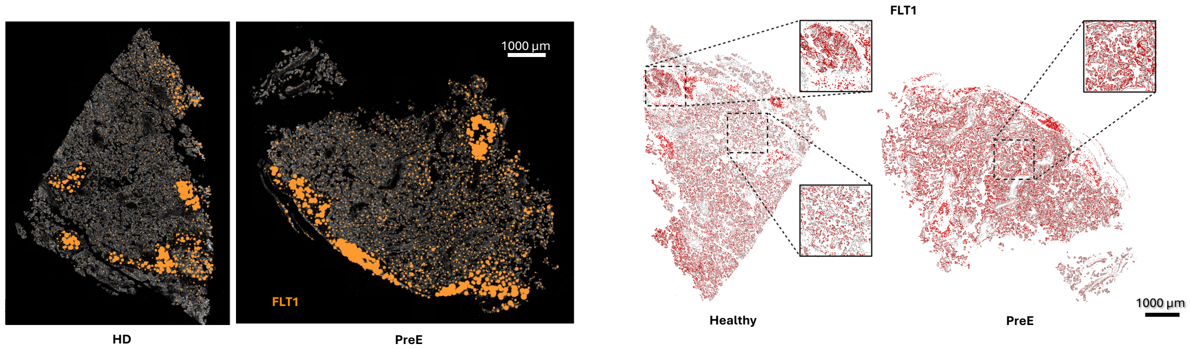

Supplemental Figure 7.

a

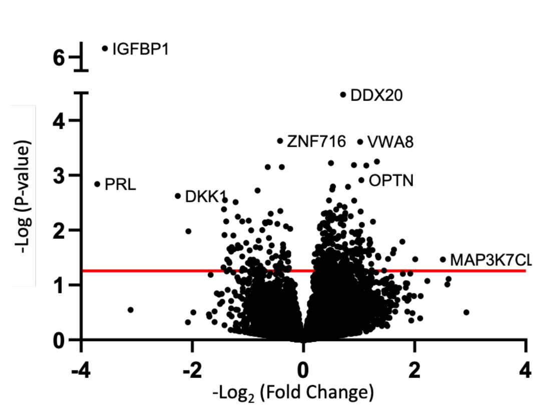

b

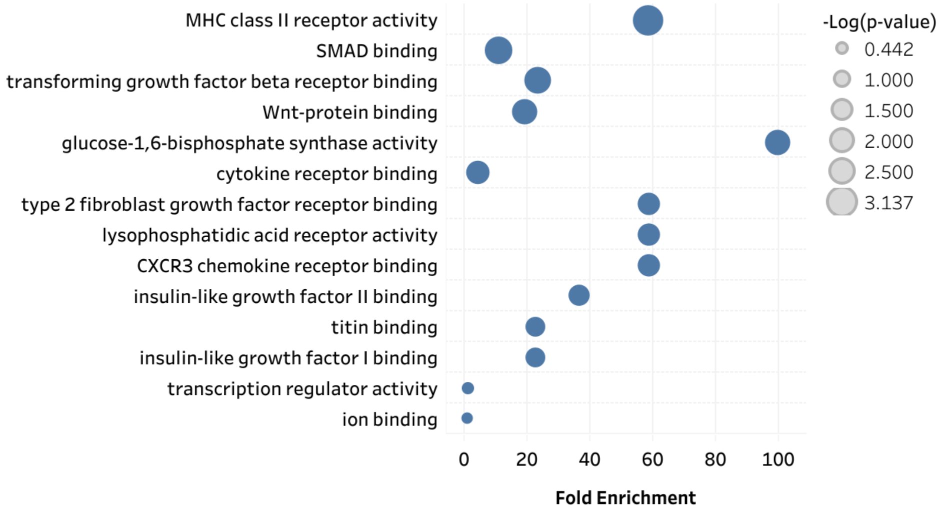

c

Predicted upstream regulator	p-value
KDM5A	0.000374
Actin	0.00059
HOXA10	0.00131
PRKCA	0.00421
HBG1	0.00518
ERBB2	0.0109
SMARCB1	0.0118
IFNGR	0.018
IL7R	0.0215
GATA5	0.0307
C9	0.0307
IL17A	0.0333
Collagen Alpha1	0.0357
PRL	0.0378
VGLL3	0.0457
ADORA2A	0.0493

d

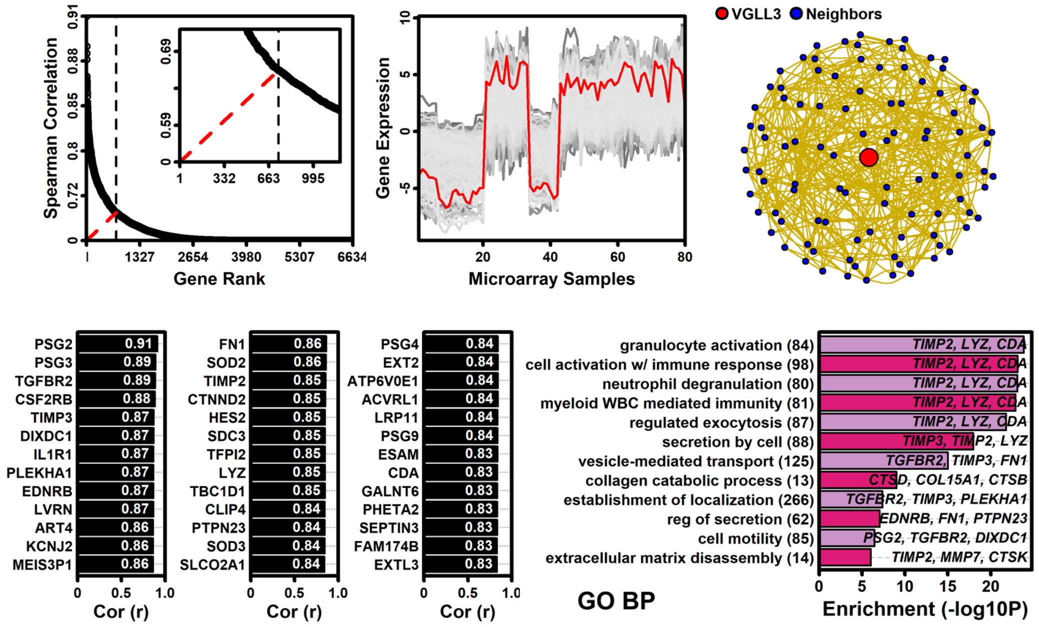

Supplemental Figure 8.

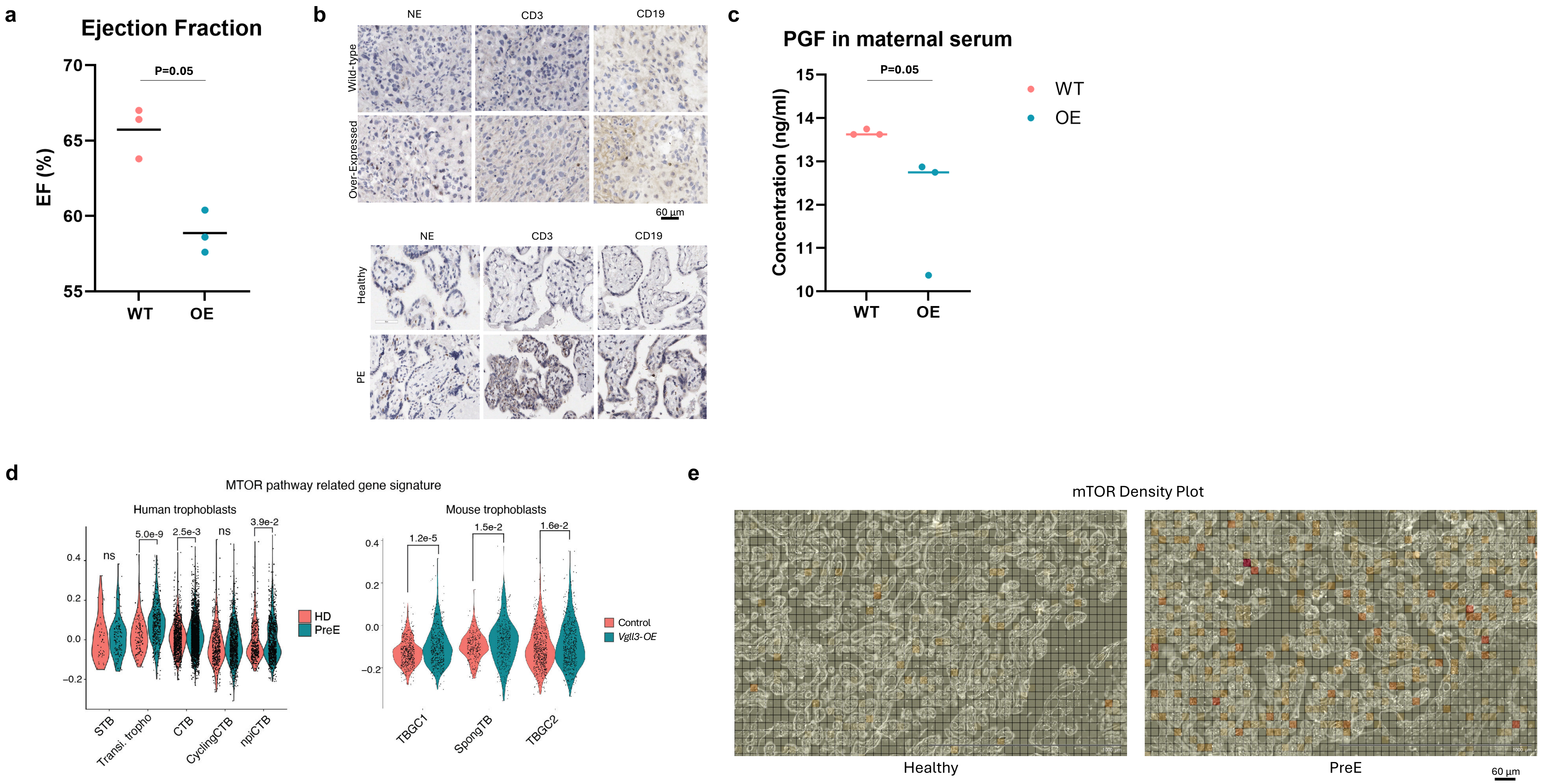

Supplemental Figure 9.

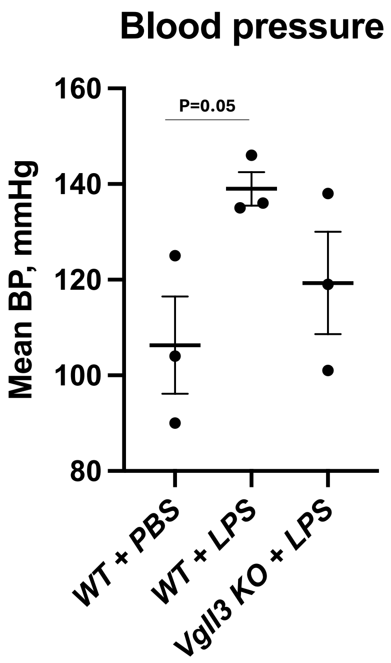

Supplemental Figure 10.

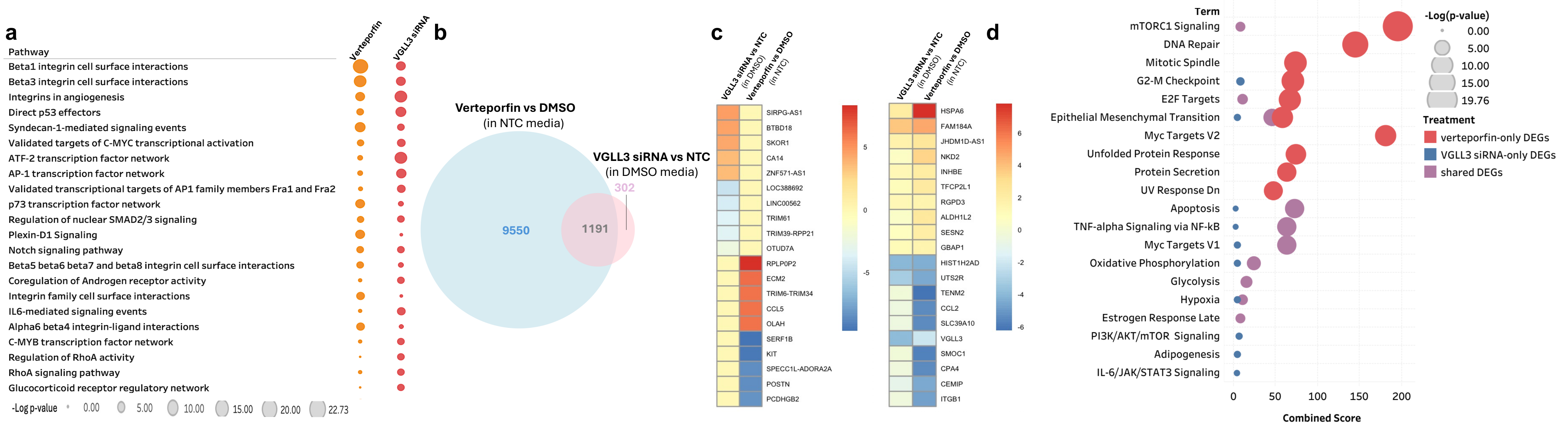

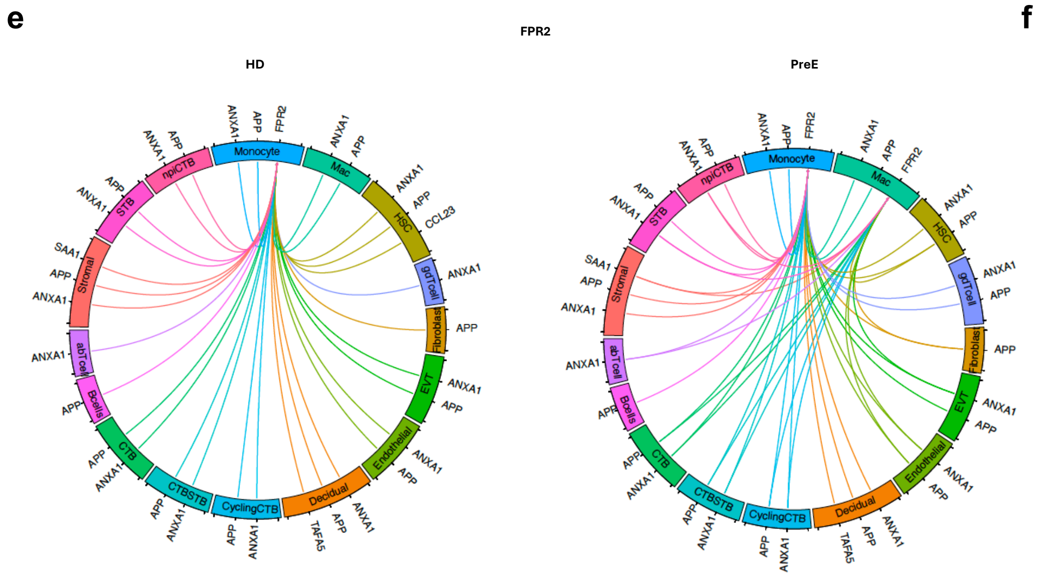

f

Verteporfin vs WRW4 in immune cells

DMSO vs Verteporfin	Common	DMSO vs WRW4
EPO signaling pathway	ErbB1 downstream signaling	IL6-mediated signaling events
Calcineurin-regulated NFAT-dependent transcription in lymphocytes	BCR signaling pathway	C-MYB transcription factor network
Angiopoietin receptor Tie2-mediated signaling	PDGFR-beta signaling pathway	Regulation of Androgen receptor activity
Validated transcriptional targets of AP1 family members Fra1 and Fra2	IL12-mediated signaling events	TCR signaling in naive CD8+ T cells
Regulation of Telomerase	Signaling events mediated by focal adhesion kinase	IL2-mediated signaling events
CXCR4-mediated signaling events	IL1-mediated signaling events	Role of Calcineurin-dependent NFAT signaling in lymphocytes
Endogenous TLR signaling	Osteopontin-mediated events	TCR signaling in naive CD4+ T cells
Validated targets of C-MYC transcriptional activation	Downstream signaling in naive CD8+ T cells	Nongenotropic Androgen signaling
Integrin-linked kinase signaling	E-cadherin signaling in the nascent adherens junction	Fc-epsilon receptor I signaling in mast cells
Cellular roles of Anthrax toxin	Canonical NF-kappaB pathway	Regulation of cytoplasmic and nuclear SMAD2/3 signaling
ATF-2 transcription factor network	CDC42 signaling events	IL12 signaling mediated by STAT4
HIF-1-alpha transcription factor network	Atypical NF-kappaB pathway	FAS (CD95) signaling pathway
Posttranslational regulation of adherens junction stability and dissassembly	RAC1 signaling pathway	
GMCSF-mediated signaling events	CD40/CD40L signaling	
RhoA signaling pathway	Ras signaling in the CD4+ TCR pathway	
Signaling events mediated by Hepatocyte Growth Factor Receptor (c-Met)	Regulation of nuclear SMAD2/3 signaling	
Signaling events mediated by VEGFR1 and VEGFR2		
Arf6 downstream pathway		
IL8- and CXCR1-mediated signaling events		
Trk receptor signaling mediated by the MAPK pathway		

Supplemental Figure 11.

a

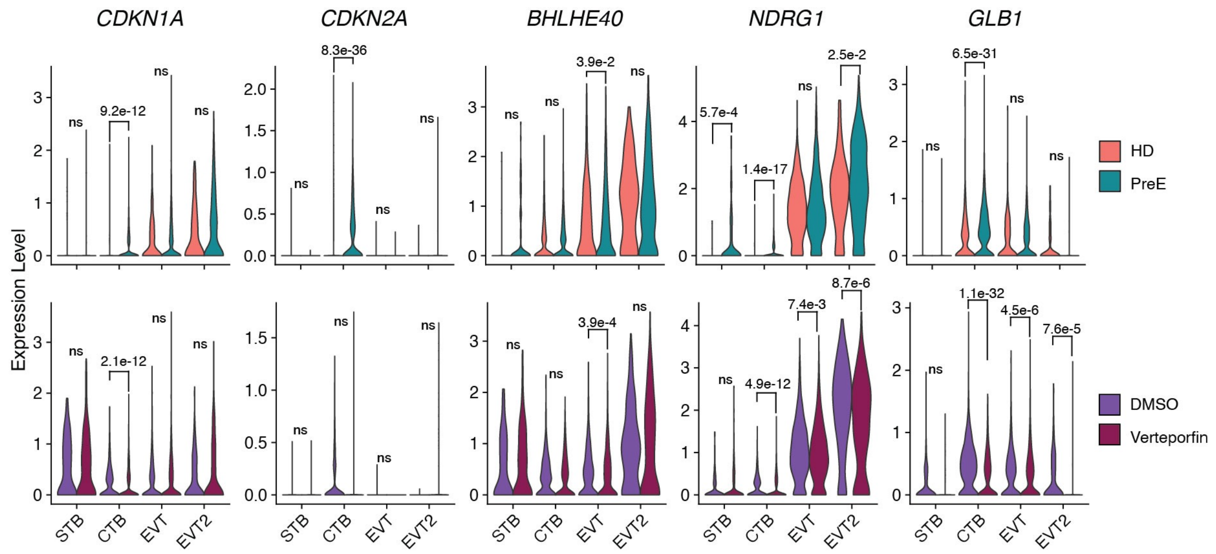

b

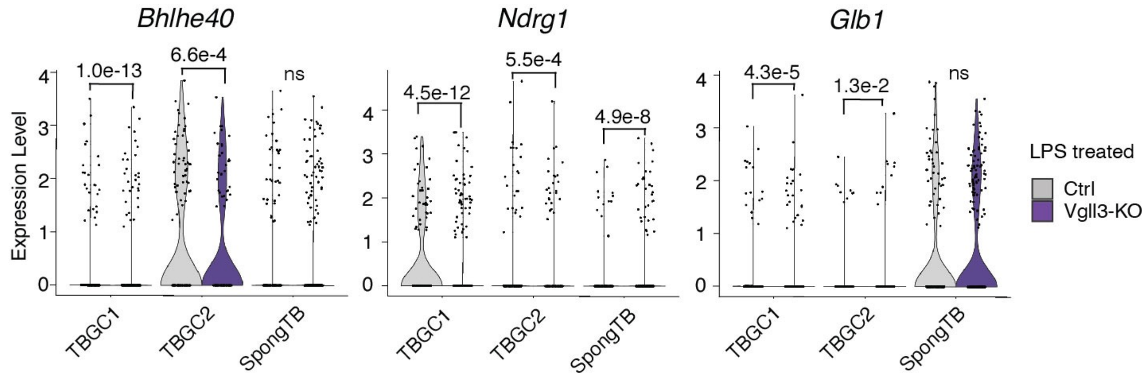

SUPPLEMENTAL TABLES

Supplemental Table 1: Cluster defining genes for human scRNA-seq (Excel file)

Supplemental Table 2: VGLL3 IP/ mass spectrometry in HEK293 cells (Excel file).

Supplemental Table 3: Cluster defining genes for mouse scRNA-seq (Excel file).

Supplemental Table 4: Genes used for module scores.

Hippo Signature	Fibrosis Signature
VGLL1	ANXA1
VGLL2	CAV1
VGLL3	CCL2
VGLL4	CCL5
TEAD1	COL3A1
TEAD2	CTGF
TEAD3	CXCL16
TEAD4	CYBB
YAP1	EGFR
WWTR1	EPAS1
WWC1	GLI1
WWC2	IGF1
WWC3	KL
LATS1	NR1H4
LATS2	PPARA
MOB1A	PPARG
SAV1	PTGS2
MAP4K4	SMAD7
MST1	TGFB1
STK3	
AMOTL1	
AMOTL2	

Supplemental Table 5: Antibodies used for immunoblotting.

Antibody target	Vendor	Catalog number	Working concentration	Secondary species	Incubation time 4C	Incubation time ambient
VGLL3	MilliporeSigma	HPA054983	1:2000	Rabbit	16-24 hours	1 hour
HA	Proteintech	66006-2-Ig	1:10000	Mouse	16-24 hours	1 hour
YY1	abcam	ab109237	1:2000	Rabbit	16-24 hours	1 hour
FLAG	Thermo	TA50011-100	1:1000	Mouse	16-24 hours	1 hour
TEAD1	SantaCruz	sc-393976	1:1000	Mouse	16-24 hours	1 hour
TEAD1	Cell Signaling	12292S	1:1000	Rabbit	16-24 hours	1 hour
turbo GFP	Thermo	TA150041	1:1000	Mouse	16-24 hours	1 hour
beta-Actin	Cell Signaling	4967L	1:1000	Rabbit	16-24 hours	1 hour

Supplemental Table 6: Antibodies used for histological staining.

Antibody target	Vendor	Catalog number	Working concentration	Retrieval buffer pH	Species stained
NE	Abcam	ab310335	1:100	pH 9	Mouse and Human
CD3	Abcam	ab215212	1:1000	pH 9	Mouse and Human
CD19	Thermo-Fisher	pa5-27442	1:250	pH 6	Mouse and Human
CD163	Abcam	ab182422	1:500	pH 9	Human
VGLL3	Millipore-Sigma	HPA054983	1:200	pH 9	Mouse and Human
FLT1	Abcam	ab2350	1:50	pH 9	Mouse and Human
TEAD1	SantaCruz	sc-393976	1:100	pH 9	Human
TEAD2	LS-Bio	LS-C342577	1:500	pH 9	Human
TEAD3	CBMAB	CBMAB-0234-LY	1:250	pH 9	Human
TEAD4	SantaCruz	sc-390578	1:200	pH 9	Human
CD11b	Invitrogen	14-0112-82	1:100	pH 6	Mouse
