# Supplementary figures and images for: Defective Trophoblast Differentiation, Endothelial Dysfunction, and Immune Dysregulation in Preeclampsia Coalesce on a Placental VGLL3-Centered Gene Network
Source: Circulation. 2026 Apr 9;153(22):1743–60. doi: 10.1161/CIRCULATIONAHA.125.076218 (PMC13120743; doi:10.1161/CIRCULATIONAHA.125.076218)

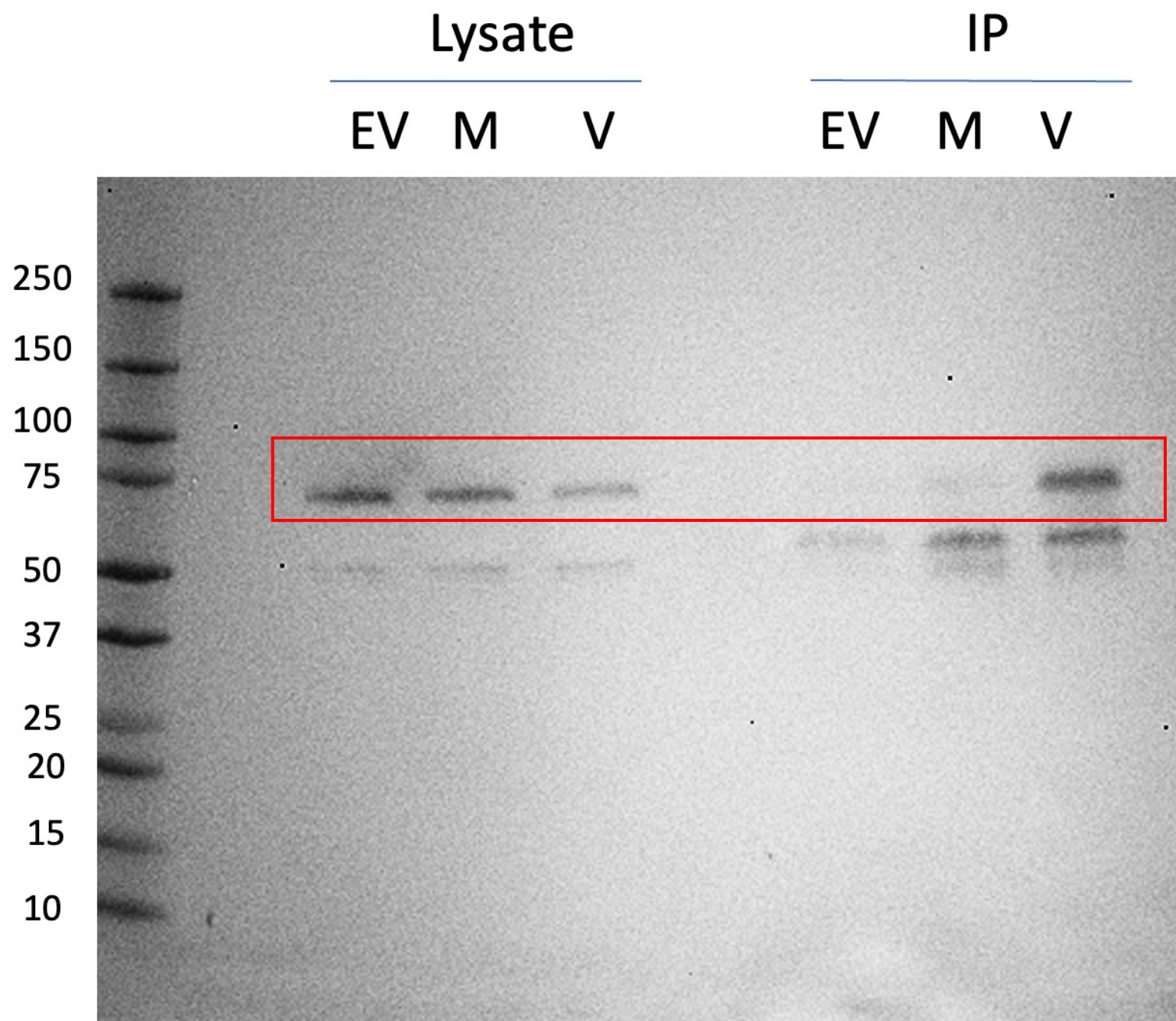

Supplement: Supplementary file 3 [file cir-153-1743-s003.pdf]
